# Ionic Liquid Catalysis in Cyclic Carbonate Synthesis for the Development of Soybean Oil-Based Non-Isocyanate Polyurethane Foams

**DOI:** 10.3390/molecules29163908

**Published:** 2024-08-18

**Authors:** Damian Kiełkiewicz, Agnieszka Siewniak, Rafał Gaida, Małgorzata Greif, Anna Chrobok

**Affiliations:** 1 Łukasiewicz Research Network—Institute of Heavy Organic Synthesis “Blachownia”, Energetyków 9, 47-225 Kędzierzyn-Koźle, Poland; damian.kielkiewicz@icso.lukasiewicz.gov.pl (D.K.); rafal.gaida@icso.lukasiewicz.gov.pl (R.G.);; 2 Department of Chemical Organic Technology and Petrochemistry, PhD School, Silesian University of Technology, Akademicka 2A, 44-100 Gliwice, Poland; 3 Department of Chemical Organic Technology and Petrochemistry, Faculty of Chemistry, Silesian University of Technology, Krzywoustego 4, 44-100 Gliwice, Poland; agnieszka.siewniak@polsl.pl

**Keywords:** non-isocyanate polyurethanes, epoxidized vegetable oils, ionic liquids, carbon dioxide

## Abstract

A method for obtaining non-isocyanate polyurethane (NIPU) foams from cyclic carbonate (CC) based on soybean oil was developed. For this purpose, cyclic carbonate was synthesized from epoxidized soybean oil and CO_2_ using various ionic liquids (ILs) as catalysts. Among the tested ILs, the highest selectivity (100%) and CC yield (98%) were achieved for 1-ethyl-3-methylimidazolium ([emim]Br). Without any purification, the resulting cyclic carbonate was reacted directly with diethylenetriamine as a model crosslinking agent to produce NIPU foams. It was found that the soybean oil-based CC synthesized with bromide imidazolium ionic liquids exhibited significantly shorter gelling times (8 min 50 s for [emim]Br and 9 min 35 s for [bmim]Br) compared to those obtained with the conventional TBAB catalyst (26 min 15 s). A shorter gelling time is a crucial parameter for the crosslinking process in foams. The obtained foams were subjected to mechanical tests and a morphology analysis.

## 1. Introduction

Polyurethanes (PUs) are versatile polymers, whose properties can be tailored across a wide range, making them suitable for numerous applications in industries from construction and automotive to footwear and furniture [1,2]. PUs are produced in various forms, including rigid and flexible foams, as well as non-porous materials such as coatings, adhesives, sealants, thermoplastics, elastomers, or thermosets for various industries. They are traditionally obtained by the polyaddition of organic aromatic or aliphatic isocyanates with compounds having at least two hydroxyl groups (polyols) [1,2,3]. For most polyurethane production, two isocyanate compounds are used: 4,4′-methylenediphenyl diisocyanate (MDI) and toluene diisocyanate (TDI). Due to their high reactivity, both MDI and TDI exhibit strong harmful and toxic properties, both in their application and in their synthesis, wherein the highly poisonous phosgene is used as a raw material [2].

An alternative to isocyanate- and polyol-based polyurethanes are linear, crosslinked non-isocyanate polyurethanes (NIPUs). They can be obtained by several methods [4,5,6,7,8,9,10,11,12,13]. One of them is polycondensation, which includes reactions between polychloroformate and polyamine, polycarbamate and polyol, polycarbamoyl chloride and polyol, polycarbonates and amino alcohols, or polycarbonate and polyamine. Among the disadvantages of this route, we can mention that the substrates used are derived from phosgene and that these reactions produce by-products, such as HCl or alcohols. The second path of NIPU synthesis includes rearrangements such as the Curtius rearrangement, Hofman rearrangement, or Lossen rearrangement. During the rearrangements, isocyanates are formed, which, like substrates such as acyl azides and carboxamides, are harmful compounds. Another route of obtaining NIPUs is subject to the ring-opening polymerization of aliphatic cyclic carbamates or aziridines. Here, phosgene is also used, mainly as a precursor to cyclic carbamates. The last method of producing NIPUs is based on the polyaddition of cyclic carbonates (CCs) with polyamines containing the primary amino groups [4,5,8,9,10]. The resulting products exhibit excellent adhesion to the different materials, due to the presence of hydroxyl groups in the chain. Polyhydroxyurethanes are also characterized by a higher thermal stability, because they do not have any thermally unstable biurets or allophanate groups in the polyurethane chain [6] but contain urethane and hydroxyl groups capable of forming intermolecular hydrogen bonds. These bonds block the carbonyl carbon atom and thereby increase the resistance to hydrolysis of the urethane group, and the chemical resistance increases twice as much compared to compounds with a similar structure but without the intermolecular hydrogen bonds [14].

Various cyclic carbonates can be used for the synthesis of non-isocyanate polyurethanes by polyaddition, including those based on petroleum-based resources. However, cyclic carbonates derived from renewable and commercially available materials, such as plant oils, terpenes, or lignin, have attracted particular attention from scientists and industry [15,16,17,18,19,20,21,22,23]. There are several methods for synthesizing cyclic carbonates [7,15,16,17,24,25]: the reaction of diols with phosgene, urea, dimethyl carbonate, or CO_2_; the reaction of CO_2_ with halohydrins; and the reaction of epoxy compounds with CO_2_. Of these methods, the reaction of epoxy compounds with CO_2_ holds the greatest practical importance. The benefit of this method is the utilization of carbon dioxide, a low-cost and sustainable feedstock [26]. Due to the low reactivity of CO_2_, catalysts are required for the reaction. The most commonly used catalysts are quaternary ammonium and phosphonium salts, organometallic catalysts, and inorganic compounds. Alternatively, ionic liquids present a promising solution due to their highly tunable structures, which enable the development of compounds with a high catalytic activity [27,28,29,30,31,32,33,34].

In addition to the low reactivity of CO_2_ with epoxides, another drawback of the NIPU foam synthesis route by polyaddition is the slow crosslinking of cyclic carbonates based on vegetable oils with polyamines. This process often requires the use of additional often harmful or toxic catalysts to accelerate the reaction or extra operations to extend the crosslinking process of the foams to ensure that a stable product is obtained. This is significant because if the reaction of the carbonate component with the polyamine is too slow, there is a loss of the blowing agent and the resulting foams collapse. Various catalysts have been proposed to accelerate the crosslinking step like 1,8-diazabicyclo[5.4.0] undec-7-ene (DBU) [35], 1,5,7-triazabicyclo[4.4.0]dec-5-ene (TBD) [36], 1-(3,5-bis(trifluoromethyl)phenyl)-3-cyclohexylthiourea [37], or 1,4-diazobicyclo[2.2.2]octane (DABCO) [38]. Another approach to increase reactivity is a combination of NIPU chemistry with other chemistries, mainly epoxy or acrylic, to produce hybrid network polymers (H-NIPUs) [39,40].

There is still a need to find efficient methods for producing NIPU foams based on vegetable oils. These methods should ensure proper crosslinking and foaming processes that occur at the same rate without the use of additional catalysts.

**Figure 1:**
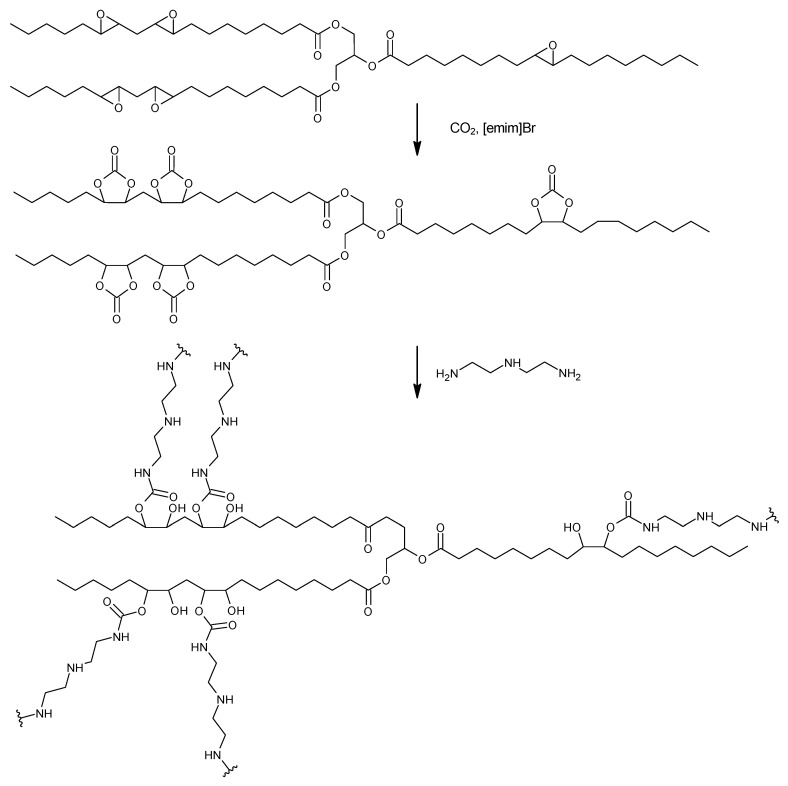
Scheme of the NIPU synthesis.

This paper evaluates the catalytic activity of various ionic liquids in the synthesis of cyclic carbonates from a soybean oil-based epoxide with CO_2_, as well as in the preparation of polyhydroxyurethane foams obtained from a soybean oil-based cyclic carbonate (CSBO) and diethylenetriamine as the model crosslinking agent (Figure 1).

## 2. Results and Discussion

### 2.1. Synthesis of Cyclic Carbonates

**Figure 2:**
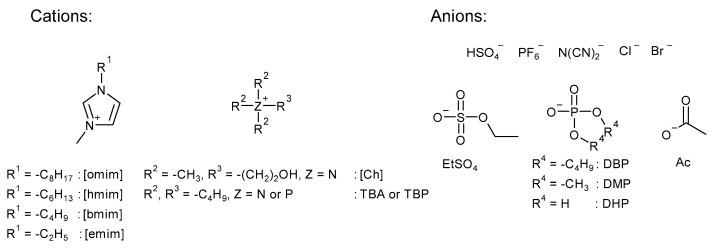
Cations and anions of ionic liquids used in this study.

A screening of ionic liquids (Figure 2) as catalysts for the reaction of epoxidized soybean oil (ESBO) with CO_2_ was conducted at 140 °C and 15 bar, using a 3 wt.% of IL relative to the epoxide.

**Figure 3:**
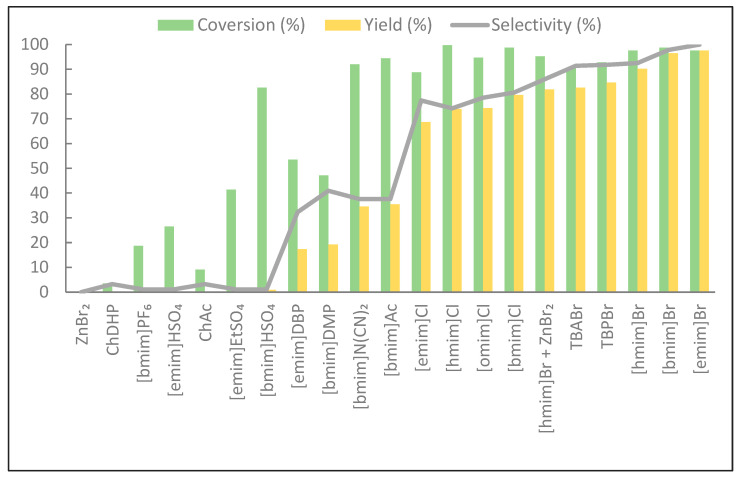
The conversion of ESBO and the selectivity and yield of CSBO in the reaction of ESBO with CO_2_ in the presence of an ionic liquid. Reaction conditions: 3 wt.% of IL, 140 °C, and 15 bar.

Among all the tested ionic liquids, the imidazolium liquids with a bromide anion were the most active (Figure 3). These liquids showed the highest activity and selectivity. The yield of cyclic carbonate with [emim]Br reached 98% with 100% selectivity. With the lengthening of the alkyl chain in the imidazolium cation and bromide anion, the yield of CC and the selectivity of the reaction decreased slightly in the following order: [emim]Br > [bmim]Br > [hmim]Br. Lower yields and selectivities were obtained for chloride-based ionic liquids. In this case, the best CC yields were obtained for [bmim]Cl and the lowest for [emim]Cl. The bromide anion is a stronger nucleophile than the chloride anion, which may facilitate its attack on the less shielded carbon atom of the epoxide and the subsequent opening of the oxirane ring. Importantly, the bromide anion also has a better leaving ability, which has a beneficial effect on the carbonate ring’s closing stage after the attachment of the CO_2_ molecule to the epoxide [41]. It is known that imidazolium ionic liquids with a bromide anion are inherently hygroscopic and readily absorb moisture from the air. We investigated the water content in [emim][Br]. For the [emim][Br] sample dried using a Schlenk line, the average water content was 0.58% (based on three measurements, each of which was 0.58%), while, for the sample frequently exposed to air, the average content was 0.85%, and we observed an increase in the water content in the IL with prolonged exposure to air. However, the presence of a certain amount of water can have a beneficial effect on catalysis and can even act as a co-catalyst. For example, Sun et al. showed that the use of water together with quaternary ammonium salt as a catalyst results in an acceleration of the cycloaddition reaction of CO_2_ to epoxides [42]. This is because water can act as a Lewis acid and, by forming a hydrogen bond between the hydrogen atom of water and the oxygen atom of the epoxide, facilitates the ring opening of the epoxide.

Based on the mechanisms described in the literature [29,30,32,42], the catalytic action of ionic liquids in the synthesis of cyclic carbonates from CO_2_ and epoxides involves the ring opening of the oxirane ring through the attack of a nucleophilic agent Br- on the less sterically hindered carbon atom of the epoxide. The water present in the system can facilitate the opening of the epoxirane ring. The resulting alkoxyl anion reacts with a CO_2_ molecule, leading to the formation of an alkyl carbonate anion, which then undergoes intramolecular substitution resulting in the formation of a cyclic carbonate, a regenerated catalyst that can take part in the next reaction cycle.

No cyclic carbonate formation was observed for protic acidic ionic liquids with acidic hydrogen on the anion such as HSO_4_, as well as for choline-based ionic liquids in which an OH group was present at one of the substituents in the quaternary ammonium cation. It is known that adding a co-catalyst such as ZnBr_2_, a Lewis acidic compound, can favorably affect the CC synthesis by making the epoxide ring more susceptible to nucleophilic attack through coordination with the oxygen atom of the epoxide. However, adding ZnBr_2_ to the catalytic system containing [hmim]Br resulted in a decreased cyclic carbonate yield and selectivity. The CC yield dropped from 90% with the ionic liquid alone to 82% with the addition of ZnBr_2_, and the selectivity decreased from 96% to 85%. The benchmark reaction with zinc bromide alone confirmed that it was not an active catalyst on its own, and the reaction mixture with its addition gelled (Table 1).

During the reaction, the viscosity of the reaction mixture increased with the conversion of the epoxide, although the greatest rise in viscosity was observed with an increase in the amount of cyclic carbonate formed, e.g., for the [emim]Br 63,800 mPa·s or for the [bmim]Br 54,500 mPa·s (Table 1).

**Figure 4:**
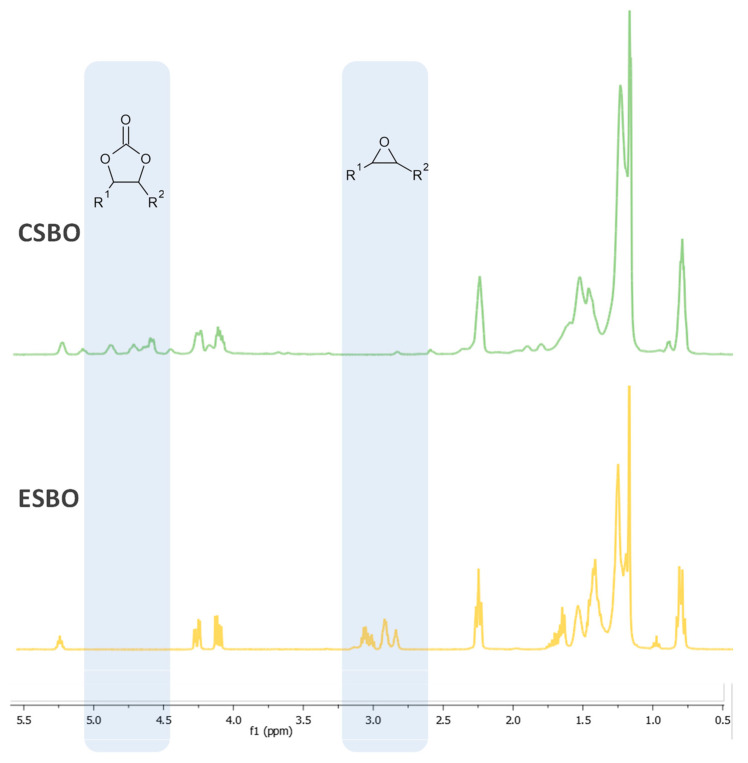
^1^H NMR spectra for ESBO and CSBO.

Comparing the ^1^H NMR spectra of ESBO and CSBO (Figure 4), the CSBO spectrum clearly shows the disappearance of peaks in a range from about 2.8 to 3.2 ppm, which are assigned to C–H bonds in the α position of the epoxy group, while the presence of new signals in a range from about 4.0 to 5.2 ppm is observed, corresponding to C–H in the α position of the cyclocarbonate group [18].

**Figure 5:**
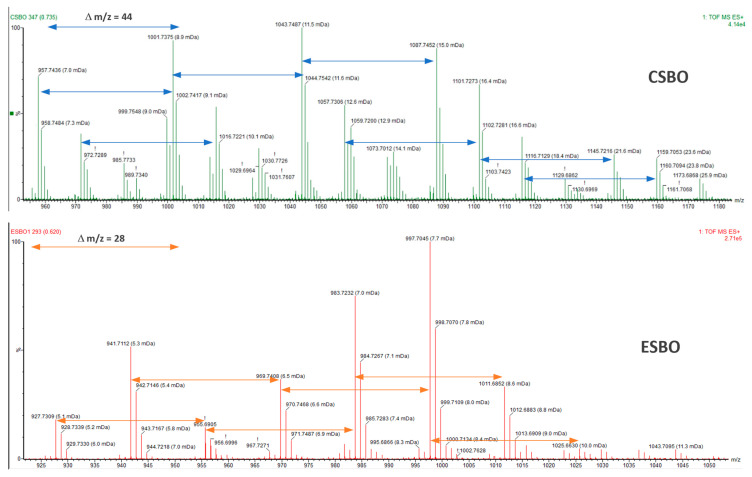
MS spectra for ESBO and CSBO.

The MS analysis of the ESBO and CSBO samples (Figure 5, Figures S35 and S36) also confirmed the formation of cyclic carbonate in the reaction of epoxide with CO_2_. Epoxidized soybean oil contains various fatty acid residues in its structure, mainly unsaturated ones with different numbers of unsaturated bonds, e.g., linoleic acid (C18) and oleic acid (C18), as well as saturated ones such as palmitic acid (C16). Hence, the spectrum contains signals between which the difference is 14 units, which corresponds to the epoxy group, or 28 units corresponding to the two methylene groups. In turn, the MS spectrum for CSBO shows signals with a mass difference of 44 u corresponding to the mass of the added CO_2_. Simultaneously, an increase in the molecular weight is observed.

**Figure 6:**
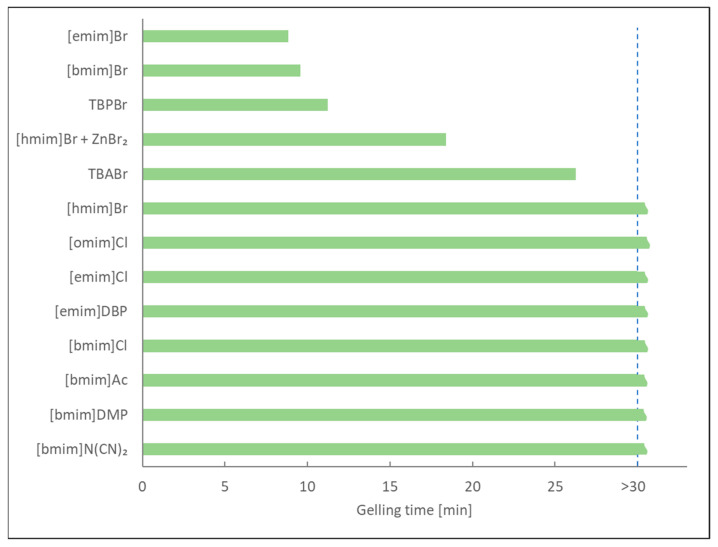
Gelling times in the reaction of CSBO with DETA in the presence of different ionic liquids as catalysts at 100 °C.

Gelling time (Figure 6), a crucial parameter for proper crosslinking in foam production, was significantly shorter for soybean oil-based CCs synthesized with bromide imidazolium ionic liquids (8 min 50 s for [emim]Br and 9 min 35 s for [bmim]Br) compared to the conventional TBAB catalyst (26 min 15 s). This shorter gelation time is critical to prevent the foam sinking during the production of NIPU foams. The reaction mixtures, without any purification, were directly reacted with diethylenetriamine as a model crosslinking agent to produce the NIPU foams.

### 2.2. Synthesis of NIPU Foams

**Figure 7:**
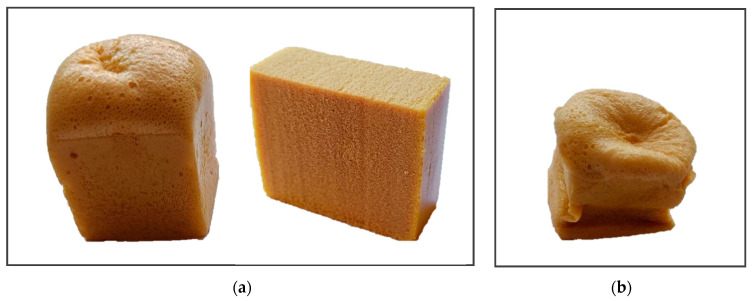
NIPU foams obtained using CSBO synthesized in the presence of (**a**) [emim]Br (IL-NIPU1) and (**b**) TBAB (TBAB-NIPU1) as catalysts.

The synthesis of NIPU foams involves a step-growth polymerization method that combines cyclic carbonates with polyamines. The main drawback of this reaction pathway is the low reactivity of these compounds, which leads to foam collapse due to the loss of the blowing agent before foam crosslinking. The reactivity strongly depends on the type of compounds used for the synthesis. The gelling time of cyclic carbonates based on primary linear glycidyl ethers with polyamines is significantly shorter than that of cyclic carbonates based on epoxidized vegetable oils, ensuring the production of stable foams. However, the low hydrolytic resistance of the former limits their application in most cases. Therefore, in our research, we focused on developing an effective method for producing oil-based NIPU foams by exploiting the difference in the gelling times of the cyclic carbonates obtained using ionic liquids as catalysts for the cycloaddition of CO_2_ to epoxides, compared to those obtained with quaternary ammonium salt. In this study, NIPU foams were synthesized from CSBO and diethylenetriamine (DETA)—a short-chain, reactive primary polyamine, with an equimolar ratio of cyclic carbonate groups to amine groups. A mixture of AZO/ZnO was used as a blowing agent and Tegostab B8406 as a surfactant. The foaming was carried out in an oven at 120 °C for 30 min, then the foam was transferred to another oven and cured for an additional 180 min at 60 °C. Figure 7 shows the foams obtained under the above conditions. The foaming of the samples occurred within the first 10 min of placing them in the oven, and, using CSBO synthesized in the presence of [emim]Br as a catalyst, a high, homogeneous foam was obtained (IL-NIPU1, Figure 7a). In contrast, the use of CSBO obtained in the presence of TBAB as a catalyst resulted in the foam collapsing after about 20 min in the oven (TBAB-NIPU1, Figure 7b).

#### 2.2.1. FT-IR Analysis

**Figure 8:**
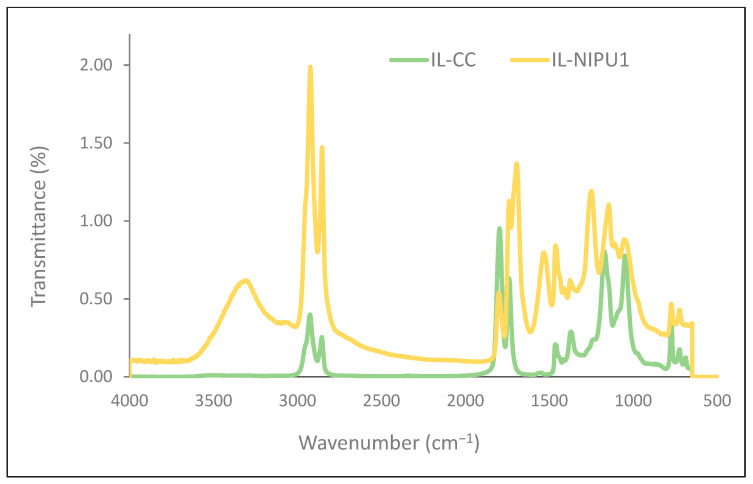
FTIR spectra of oil-based cyclic carbonate and resulting polyhydroxyurethane foam (IL-NIPU1).

An infrared spectroscopy analysis was performed to check the conversion of the carbonate groups of CSBO with amine groups of DETA. Figure 8 presents the FTIR spectra of oil-based cyclic carbonate (IL-CC) and the resulting polyhydroxyurethane foam (IL-NIPU1). The characteristic stretching vibration of the cyclic carbonate carbonyl group (C=O) at 1800 cm^−1^ diminishes, indicating that the cyclic carbonate is consumed in the process of forming the urethane linkage. Concurrently, new bands appear, including a broad band between 3500 and 3100 cm^−1^, which is indicative of N-H stretching vibrations from the urethane groups and hydroxyl groups, attributed to the ring-opening reaction of cyclic carbonate by amine. Additionally, a new band at 1694 cm^−1^ assigned to the urethane carbonyl (C=O) stretching vibration and an N-H bending vibration at 1532 cm^−1^; confirms the formation of urethane linkages in the polymer structure.

#### 2.2.2. Amount of Blowing Agent

**Figure 9:**
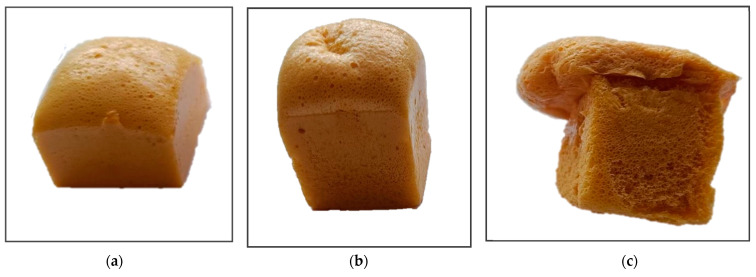
IL-NIPU foams obtained with various amounts of the blowing agent: (**a**) 6.5% (IL-NIPU2); (**b**) 10% (IL-NIPU1); and (**c**) 13.5% (IL-NIPU3).

Using the IL-CSBO, foaming optimization trials with DETA as a curing agent were conducted, varying the amount of the blowing agent. Figure 9 presents the foams obtained with 6.5% (IL-NIPU2), 10% (Il-NIPU1), and 13.5% (Il-NIPU3) of AZO/ZnO, related to the amount of CSBO in the composition. The foam IL-NIPU2, obtained with the smallest amount of the blowing agent, was characterized with the lowest height and had a density of 440 kg/m^3^. The foam produced using a blowing agent in the amount of 10% (IL-NIPU1) was homogeneous and had a density of 235 kg/m^3^ with even, small pores. Meanwhile, the foam obtained in the presence of 13.5% of the blowing agent overflowed from the mold and had large, uneven pores, especially in the lower part. Due to its non-uniform structure, it was not possible to determine its density.

#### 2.2.3. Mechanical Tests

The specimen IL-NIPU1 was cut to a size of 50 × 50 mm and a thickness of 25 mm, and, using the Instron 4466 testing machine (Instron, Norwood, MA, USA), the foam was analyzed according to the method [43]. The compression force deflection (CFD) and hysteresis loss of the NIPU foams were determined as stress at 50% of the compression strain. This test consists of measuring the force necessary to produce designated indentations in the foam product.

The hysteresis loss and elastic recovery were calculated as follows:

$$Hysteresis\;loss\;\left(\%\right)=\frac{loading\;energy-unloading\;energy}{loading\;energy}\times 100\%$$

$$Elastic\;recovery\;\left(\%\right)=\frac{compression\;strain-residual\;strain}{compression\;strain}\times 100\%$$

**Figure 10:**
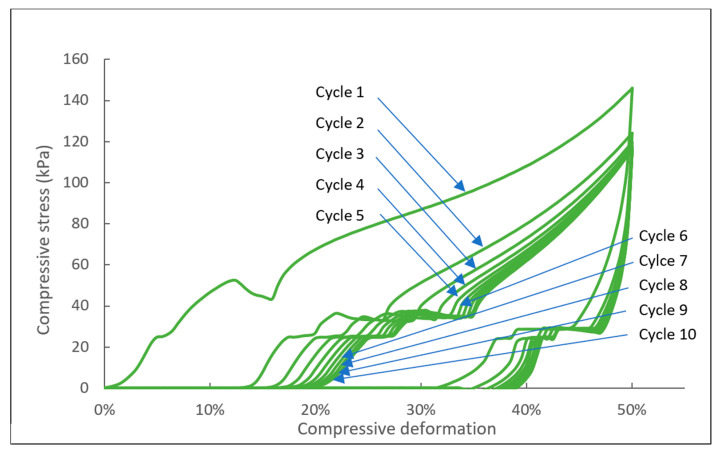
Compression curves of IL-NIPU1 foam.

The CFD curves of the IL-NIPU1 foam were determined by compressing the sample in ten cycles with a compression rate of 50 mm/min. The loading energy determined in the first cycle was 1338 mJ, the unloading energy was 198 mJ, and the hysteresis loss was 85%. In the tenth cycle, these values were represented by 540 mJ, 102 mJ, and 81.1%, respectively. The elastic recovery after the first cycle was 75.5% and, after the last one, was 63.1%. The biggest elastic recovery loss was observed after the first cycle; in subsequent cycles, the loss was much smaller. Importantly, after some time, the foams returned to their initial height. The results are presented in Figure 10 and Table 2. The platform region in the compression curves of the IL-NIPU1 foam demonstrates its energy absorption capabilities, making it suitable for impact resistance or cushioning applications. This region reflects the foam’s structural stability, as its open-cell morphology with smooth, uniform walls helps distribute stress evenly, leading to a stable compression response. The material behavior in this region shows a balance between the initial resistance to deformation and subsequent compliance, which is behavior typical of materials that combine both flexible and rigid characteristics.

**Figure 11:**
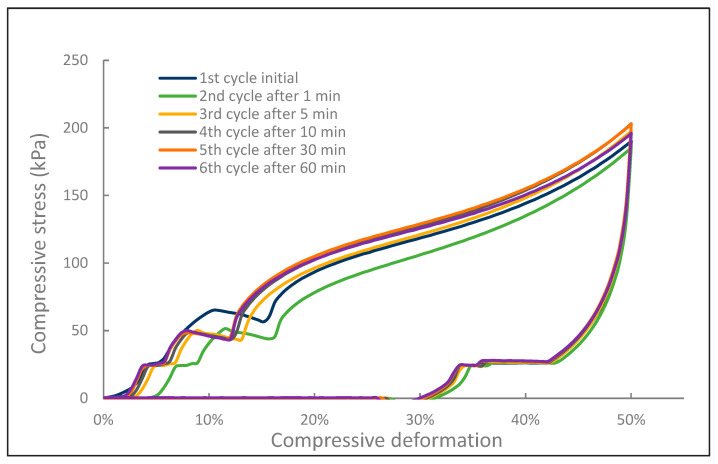
Compression curves of IL-NIPU1 foam interval stress test.

In another test, the IL-NIPU1 was initially compressed two times to 50% of its original thickness with a compression rate of 50 mm/min and left for 6 min to eliminate any residual strains after the reaction. Then, the actual measurement was made by compressing and relaxing the foam to obtain its starting conditions, and then it was repeated in loops after 1, 5, 10, 30, and 60 min with the same compression rate. The determined stress values at a 50% strain were 190, 185, 197, 203, 202, and 195 kPa, respectively. The results are shown in Figure 11.

In conclusion, the tested foam exhibited properties typical of both flexible and rigid foams. During the first cycle, the stress required to compress the foam was much higher than in subsequent cycles and the return to the original height took longer than in the case of standard flexible foams. However, no crumbling was observed and the foam remained completely homogeneous.

#### 2.2.4. Thermal Stability

**Figure 12:**
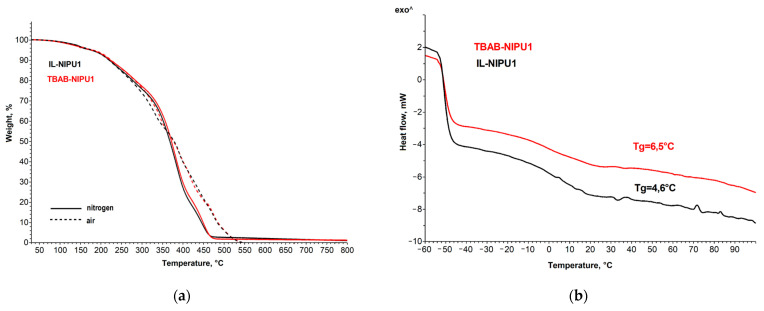
(**a**) TG curves of NIPU samples in nitrogen (solid line) and air (dotted line); and (**b**) DSC curves of NIPU samples with the determined glass transition temperature (Tg).

The thermal stability of the NIPU samples was investigated by a TGA analysis in nitrogen (an inert atmosphere) and the thermoxidative stability being tested in air (an oxidative atmosphere). The TG curves are shown in Figure 12a. From the thermogram, it is clearly observed that the IL-NIPU1 and TBAB-NIPU1 samples exhibit an identical thermal and thermooxidative stability. Their thermal properties are the same, which may suggest a very similar chemical structure. Furthermore, the glass transition temperatures (Tg) of these samples are likewise comparable (4.6 °C for IL-NIPU1 and 6.5 °C for TBAB-NIPU1)—Figure 12b.

**Figure 13:**
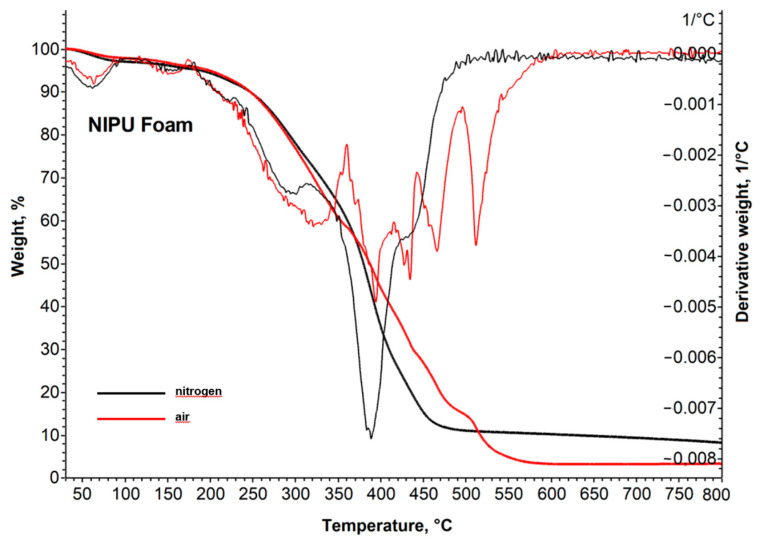
The TG and DTG curves of the NIPU foam.

The thermogravimetric decomposition of NIPU foam in nitrogen and air is shown in Figure 13. When the temperature exceeded 180 °C, a significant weight loss occurred, indicating the breakdown of the polymer backbone. The NIPU foam does not exhibit a high degree of thermal stability, as a 5% weight loss was observed at approximately 190 °C. However, it exhibits low char formation and residual mass, with values around 1.9% and 8.2% at 800 °C, respectively.

#### 2.2.5. Influence of Solvents

**Figure 14:**
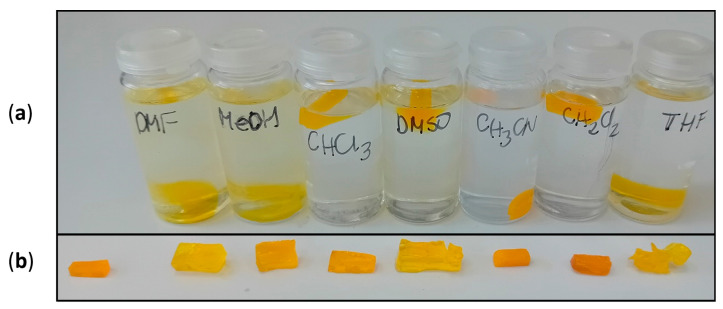
(**a**) The appearance of IL-NIPU1 in solvents after exposure to the following conditions: an ultrasound at a temperature of 50 °C for 10 h; and (**b**) the appearance of IL-NIPU1 after removing it from the solvents. On the left is a reference piece of NIPU.

The IL-NIPU1 was subjected to solvent treatment under harsh conditions: an ultrasound at a temperature of 50 °C for 10 h. In no case was the dissolution of NIPU observed. Only the DMF and methanol slightly stained it. In solvents such as DMF, methanol, DMSO, THF, and, to a small degree, chloroform, it swelled (Figure 14).

#### 2.2.6. Morphology Analysis

**Figure 15:**
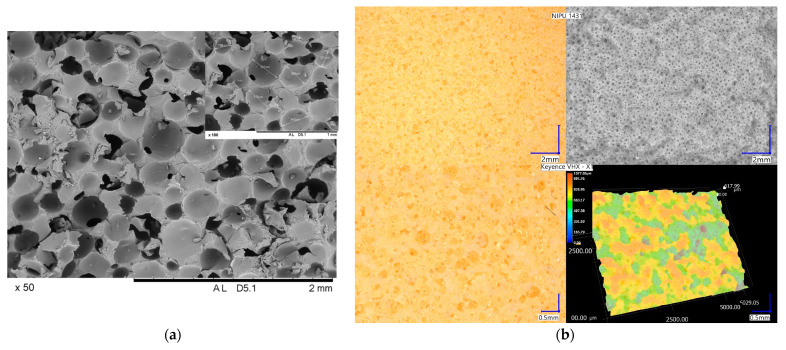
(**a**) SEM image of IL-NIPU1 polyhydroxyurethane foam; and (**b**) a photo of IL-NIPU1 foam taken with a Keyence VXH-X1 digital microscope. Photo taken courtesy of Keyence.

The physical characteristics of foams are influenced not only by the polymer matrix stiffness but also by the foam cell structure. The morphology of the IL-NIPU1 foam was investigated by the SEM method at a magnification of 50 and 100. Figure 15a shows a mainly open-cell structure with a good homogeneity. The cells are cylindrical with a typical diameter in the range of 150–300 µm and are characterized by very smooth, uniform walls. No aggregates or inhomogeneities are visible. Figure 15b shows the foam surface using a digital microscope.

## 3. Materials and Methods

### 3.1. Materials

The epoxidized soybean oil (ESBO) and azodicarbonamide (AZO, Unifoam AZ) were obtained from Krahn Chemie Polska (Poznań, Poland); the carbon dioxide was supplied by Air Products (Poland); and the diethylenetriamine (DETA), tetrabutylammonium bromide (TBAB), zinc bromide (ZnBr_2_), and zinc oxide (ZnO) were purchased from Sigma-Aldrich. The ionic liquids were provided by IoLiTec Ionic Liquids Technologies GmbH (Raaba-Grambach, Austria). These ionic liquids were purchased from IoLiTec Ionic Liquids Technologies GmbH: 1-butyl-3-methylimidazolium hydrogen sulphate ([bmim]HSO_4_), 1-butyl-3-methylimidazolium hexafluorophosphate ([bmim]PF_6_), 1-butyl-3-methylimidazolium dicyanamide ([bmim]N(CN)_2_), 1-butyl-3-methylimidazolium dimethylphosphate ([bmim]DMP), 1-butyl-3-methylimidazolum acetate ([bmim]Ac), 1-butyl-3-methylimidazolium bromide ([bmim]Br), 1-butyl-3-methylimidazolium chloride ([bmim]Cl), 1-ethyl-3-methylimidazolium ethylsulphate ([emim]EtSO_4_), 1-ethyl-3-methylimidazolium dibutylphosphate ([emim]DBP), 1-ethyl-3-methylimidazolium hydrogen sulphate ([emim]HSO_4_), 1-ethyl-3-methylimidazolium bromide ([emim]Br), 1-ethyl-3-methylimidazolium chloride ([emim]Cl), 1-hexyl-3-methylimidazolium chloride ([hmim]Cl), 1-octyl-3-methylimidazolium chloride ([omim]Cl), 1-hexyl-3-methylimidazolium bromide ([hmim]Br), tetrabutylphosphonium bromide (TBPBr), choline dihydrophosphate (ChDHP), choline acetate (ChAc), and Tegostab B8406 (Evonic). All of the materials were used as received without any further purification.

### 3.2. Analysis of Materials

The epoxy value was determined according to the standard PN-EN ISO 3001 [44] by titration method. The EV for ESBO = 0.376 mol/100 g. The viscosity of the oils was measured at 25 °C using a Brookfield DV II viscometer, according to PN-86/C-89085/06 [45]. The gelling times were measured using a Coesfeld Geltest GT16, by mixing stoichiometric amounts of CSBO with DETA at 100 °C, according to PN-C-89085-19. The mechanical properties of the foams were determined using an Instron 4466 testing machine according to the ASTM D3574-05 method [43] with 50% strains, instead of 75%. The progress of carbonization reaction was characterized using the FT-IR method. The presence of cyclic carbonate groups in the tested samples was confirmed using the FT-IR method, based on the standard [46] PN-ISO 6286 with the use of an FT-IR THERMO Scientific Nicolet 6700 spectrometer equipped with a SMART ARK ATR attachment, supported with the Omnic and TQ Analyst software. The ATR reflection spectra for the samples directly spread on the plate were recorded in the range of 4000–650 cm^−1^, with a resolution of 4 cm^−1^ and 64 scans. The SEM images of the synthesized foams were performed applying an equipped TM3000 SEM instrument (10 kV) (Hitachi Tokyo, Japan). The photos of the foams were taken with a VXH-X1 digital microscope (Keyence International, Mechelen, Belgum). The high-resolution mass spectrometry analyses were performed on a Waters Xevo G2 Q-TOF mass spectrometer (Waters Corporation, Milford, CT, USA) equipped with an ESI source operating in the positive ion mode. The full-scan MS data were collected from 200 to 2000 Da in the positive ion mode with a scan time of 0.1 s. The data were collected in the centroid mode, and the mass was corrected during acquisition using a leucine enkephalin solution as an external reference (Lock-Spray TM; *m*/*z* 556.2771 Da ([M + H]^+^) in positive ESI mode). The scanning electron microscopy was performed on a Phenom Pro Desktop SEM. The NMR spectroscopy was performed on a Varian 400 MHz NMR system. The thermogravimetric analyses (TGAs) were conducted using a Mettler Toledo thermobalance. The DSC analysis was performed using a Mettler Toledo DSC 822 scanning calorimeter with the DSC measurements for ~10 mg of the analytical sample taking place in a 40 µL Al measuring vessel with a perforated lid (1 mm) in a dynamic (50 mL·min^−1^) nitrogen atmosphere. The water content was determined using a Karl Fischer 915 KF Ti-Touch titration kit (Metrohm, Herisau, Switzerland).

Based on the height and surface areas of the bands at wavelengths of 850 and 910 cm^−1^, characteristic for epoxy groups, and the band at a wavelength of 1800 cm^−1^, characteristic for cyclic carbonate groups, numerical data characterizing the content of the epoxy and carbonate groups in the obtained products were determined. These data allowed for the determination of the conversion of the epoxy groups, measured by their depletion, and the selectivity of the reaction to cyclic carbonate groups.

The conversion of epoxy groups was calculated according to the following formula:

$$\frac{\%{EP}_{p}-\%{EP}_{k}}{\%{EP}_{p}}\times 100$$

where

%*EP_p_*—the content of epoxy groups in the raw epoxidized oil; and %*EP_k_*—the content of epoxy groups in the product.

The selectivity of the conversion of epoxy groups to cyclic carbonate groups in the products was calculated according to the following formula:

$$\frac{\%CC}{\%CC+\%EP}\times 100$$

where

%*CC*—the content of cyclic carbonate groups in the product; and %*EP*—the content of epoxy groups in the product.

The reaction yield was calculated according to the following formula:

$$\frac{conversion\;of\;the\;epoxy\;groups\times selectivity}{100}$$

### 3.3. Procedure for Cycloaddition of CO_2_ to Epoxidized Vegetable Oil

The cyclic carbonate was prepared by the cycloaddition reaction of ESBO with carbon dioxide in the presence of the catalysts. Epoxidized soybean (200 g) and a catalyst (6 g) were charged into a 300 mL stainless steel autoclave (Autoclave Engineers 300 mL) equipped with a gas inlet tube, sample tube, mechanical stirrer, and thermometer; heated to 140 °C; and then pressurized to 15 bar. The reaction was carried out to the almost complete conversion of the epoxy groups to cyclic carbonate groups, and samples were taken after 4.5, 12, 19, 26, and 33 h of mixing. The obtained products were crosslinked and foamed without any purification. Each experiment was repeated two times, and the average value was taken as the result.

### 3.4. Procedure for Synthesis of NIPU Foams

The NIPU foams were synthesized in silicone molds according to the following procedure: CSBO (30 g), polyether polydimethylsiloxane copolymer Tegostab B8406 as a surfactant (0.3 g), and a mixture of AZO/ZnO 75%/25% as a blowing agent in the different amounts were stirred mechanically at 2000 rpm for 2 min at room temperature. Then, DETA (6 g) as a crosslinker was added and stirred for another 3 min at 2000 rpm till a homogeneous mixture was obtained. The mold was placed inside the oven where the foaming and curing took place at 120 °C for 30 min and then for an additional 180 min at 60 °C. The compositions of the formulations used in the NIPU synthesis are given in Table 3.

## 4. Conclusions

An efficient method for obtaining NIPU foams from epoxidized soybean oil was developed. The method consists of two stages. The first stage involves the cycloaddition reaction of CO_2_ to the epoxidized soybean oil, and, in the second stage, the resulting cyclic carbonate reacts with diethylenetriamine in the presence of a blowing agent. The first step was carried out in the presence of a 3 wt.% of IL at 140 °C and under 15 bar of CO_2_ pressure. It was found that, among the catalysts tested, imidazolium ionic liquids with bromide anion were the most active. In the case of [emim]Br, cyclic carbonate was obtained with 100% selectivity and a 98% yield. Moreover, the gelation time for this IL was about 18 min shorter than for the conventional TBAB catalyst. Gelling time is a critical parameter in foam production. A shorter gelling time prevents the loss of a blowing agent, thereby reducing the risk of foam collapse. After the reaction, the cyclic carbonate was directly used to prepare NIPU foams. The cyclic carbonate was reacted with diethylenetriamine in the presence of a mixture of AZO/ZnO as a blowing agent and Tegostab B8406 as a surfactant. The key finding is that imidazolium bromide is a suitable, highly active catalyst for both steps in obtaining the NIPU foams. This means that there is no need to purify the obtained cyclic carbonate before sending it to foam production and no need for an additional catalyst in the second stage. This simplifies the procedure for obtaining foams. The developed method is more sustainable because it uses renewable resources and captures CO_2_, thus contributing to a reduction in greenhouse gas emissions. Instead of cyclic carbonates derived from petroleum, vegetable oil-based carbonates are used, making the method more environmentally friendly. In particular, compared to the traditionally synthesized foams of organic aromatic or aliphatic isocyanates with compounds, no harmful compounds are used. In addition, the developed method allows the production of NIPU foams with comparable mechanical properties.

## Figures and Tables

**Table 1:** Epoxy value (mol/100 g) change during the reaction of ESBO with CO_2_ in the presence of ionic liquid. Reaction conditions: 3 wt.% of IL, 140 °C, and 15 bar; mol% of catalysts in relation to ESBO.

Catalyst	% mol of Catalyst	Reaction Time/EV [h/(mol/100 g)]					Viscosity [mPa·s]
		4.5	12	19	26	33	
[bmim]HSO_4_	12.37	0.336	0.283	0.189	0.105	0.065	−
[bmim]PF_6_	10.28	0.318	0.316	0.313	0.306	0.304	−
[bmim]N(CN)_2_	14.24	0.353	0.301	0.237	0.111	0.030	83,700
[bmim]DMP	11.06	0.355	0.312	0.269	0.238	0.198	6100
[bmim]Ac	14.74	0.338	0.134	0.071	0.021	−	28,800
[bmim]Br	13.34	0.307	0.237	0.107	0.005	−	54,500
[bmim]Cl	16.73	0.091	0.017	0.005	−	−	44,400
[emim]Cl	19.93	0.246	0.126	0.042	0.005	−	39,200
[hmim]Cl	14.41	0.124	0.076	0.001	−	−	37,200
[omim]Cl	12.66	0.212	0.080	0.020	0.009	−	38,900
[emim]EtSO_4_	12.37	0.363	0.329	0.317	0.243	0.219	−
[emim]DBP	9.12	0.322	0.314	0.263	0.212	0.174	17,500
[emim]HSO_4_	14.03	0.348	0.340	0.300	0.275	0.232	1460
[emim]Br	15.29	0.305	0.215	0.101	0.009	−	63,800
[hmim]Br	11.82	0.321	0.099	0.009	−	−	29,800
[hmim]Br + ZnBr_2_	12.64	0.317	0.185	0.046	0.018	−	21,100
ZnBr_2_	12.98	0.231	0.222	0.200	−	−	gelled
ChDHP	14.53	0.362	0.361	0.361	−	−	−
ChAc	17.90	0.340	0.340	−	−	−	−
THPBr	6.47	0.237	0.150	0.056	0.036	−	40,100
TBABr	9.06	0.301	0.198	0.054	0.027	−	30,800

**Table 2:** CFG and elastic recovery of IL-NIPU1 foam compressed in 10 cycles.

Cycle Number	CFD (kPa)	Elastic Recovery (%)
1	146	100.0
2	124	75.5
3	120	70.9
4	118	68.1
5	117	66.9
6	116	66.0
7	116	65.0
8	116	64.1
9	116	64.0
10	116	63.1

**Table 3:** Composition of the formulations used in the NIPU synthesis.

Component	IL-NIPU1 [g]	IL-NIPU1 [wt%]	IL-NIPU2 [g]	IL-NIPU2 [wt%]	IL-NIPU3 [g]	IL-NIPU3 [wt%]
IL-CSBO	30.00	78.43	30.00	76.34	30.00	74.35
Tegostab B8406 (surfactant)	0.30	0.78	0.30	0.76	0.30	0.74
AZO/ZnO (blowing agent)	1.95	5.10	3.00	7.63	4,05	10.04
DETA	6.00	15.69	6.00	15.27	6.00	14.87

## Data Availability

Dataset available on request from the authors.

## Supplementary Materials

The following supporting information can be downloaded at: https://www.mdpi.com/article/10.3390/molecules29163908/s1, Figures S1–S34: NMR spectra of ESBO, CSBO and ionic liquids; Figures S35–S37: ESI-MS spectra of ESBO and CSBO; Figures S38–S40: TGA and DSC curves of IL-NIPU1 and TBAB-NIPU1; Figure S41: FT-IR spectrum of DETA; Figure S42: FT-IR spectra of CSBO obtained using different IL catalysts.
