# Supplementary material for: Ionic Liquid Catalysis in Cyclic Carbonate Synthesis for the Development of Soybean Oil-Based Non-Isocyanate Polyurethane Foams
Source: Molecules. 2024 Aug 18;29(16):3908. doi: 10.3390/molecules29163908 (PMC11356965; doi:10.3390/molecules29163908)

Supplementary Materials

Ionic liquid catalysis in cyclic carbonate synthesis for the development of soybean oil-based non-isocyanate polyurethane foams

Damian Kiełkiewicz [1,2], Agnieszka Siewniak [3], Rafał Gaida [1], Małgorzata Greif [1], and Anna Chrobok[1,*]

[1] Łukasiewicz Research Network - Institute of Heavy Organic Synthesis "Blachownia", Energetyków 9, 47-225 Kędzierzyn-Koźle; damian.kielkiewicz@icso.lukasiewicz.gov.pl; rafal.gaida@icso.lukasiewicz.gov.pl; malgorzata.greif@icso.lukasiewicz.gov.pl.
[2] Department of Chemical Organic Technology and Petrochemistry, PhD School, Silesian University of Technology, Akademicka 2A, 44-100 Gliwice, Poland
[3] Department of Chemical Organic Technology and Petrochemistry, Faculty of Chemistry, Silesian University of Technology, Krzywoustego 4, 44-100 Gliwice, Poland; agnieszka.siewniak@polsl.pl
* Correspondence: anna.chrobok@polsl.pl.

Contents:

Fig. S1-33 NMR spectra of ESBO, CSBO and ionic liquids.

Fig. S34-37 ESI-MS spectra of ESBO and CSBO.

Fig. S38-40 TGA and DSC curves of IL-NIPU1 and TBAB-NIPU1.

Fig. S41 FT-IR spectrum of DETA.

Fig. S42 FT-IR spectra of CSBO obtained using different IL catalysts.

Fig. S1 ^{1}H NMR spectrum of ESBO.

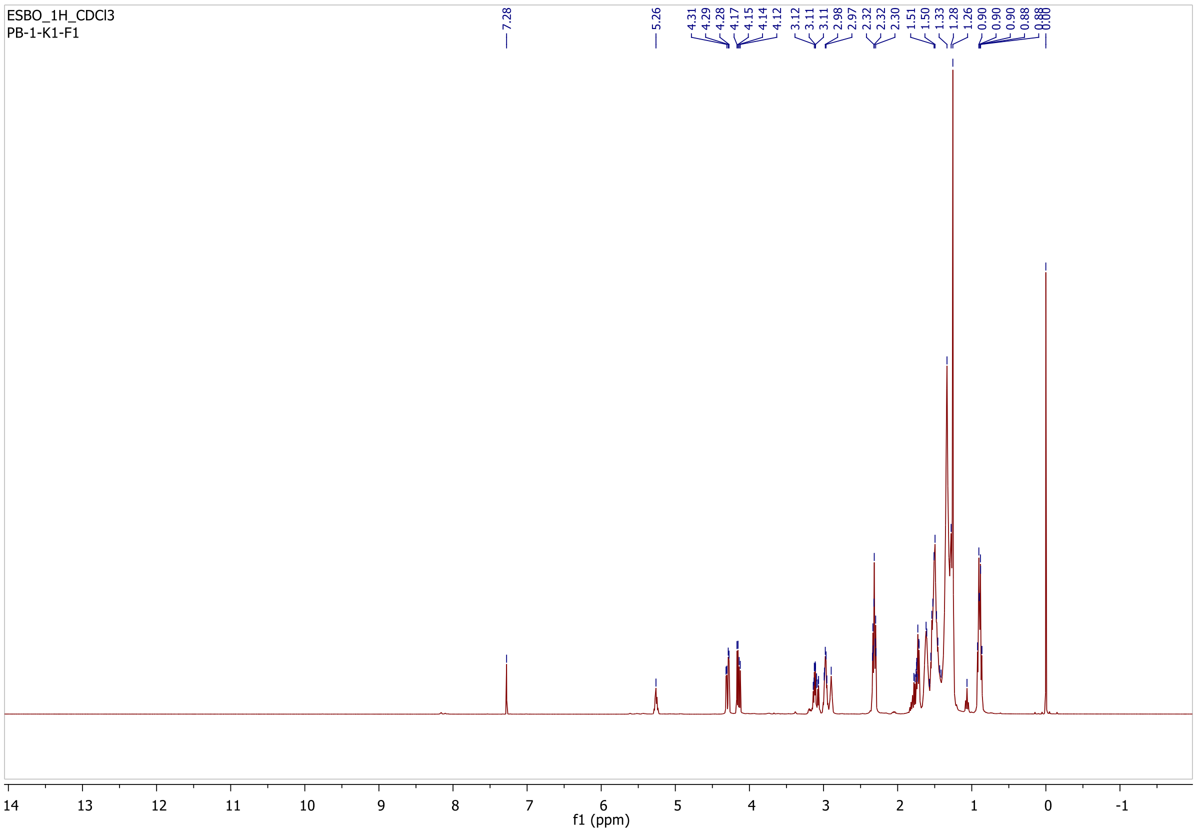

Fig. S2 ^{1}H NMR spectrum of CSBO.

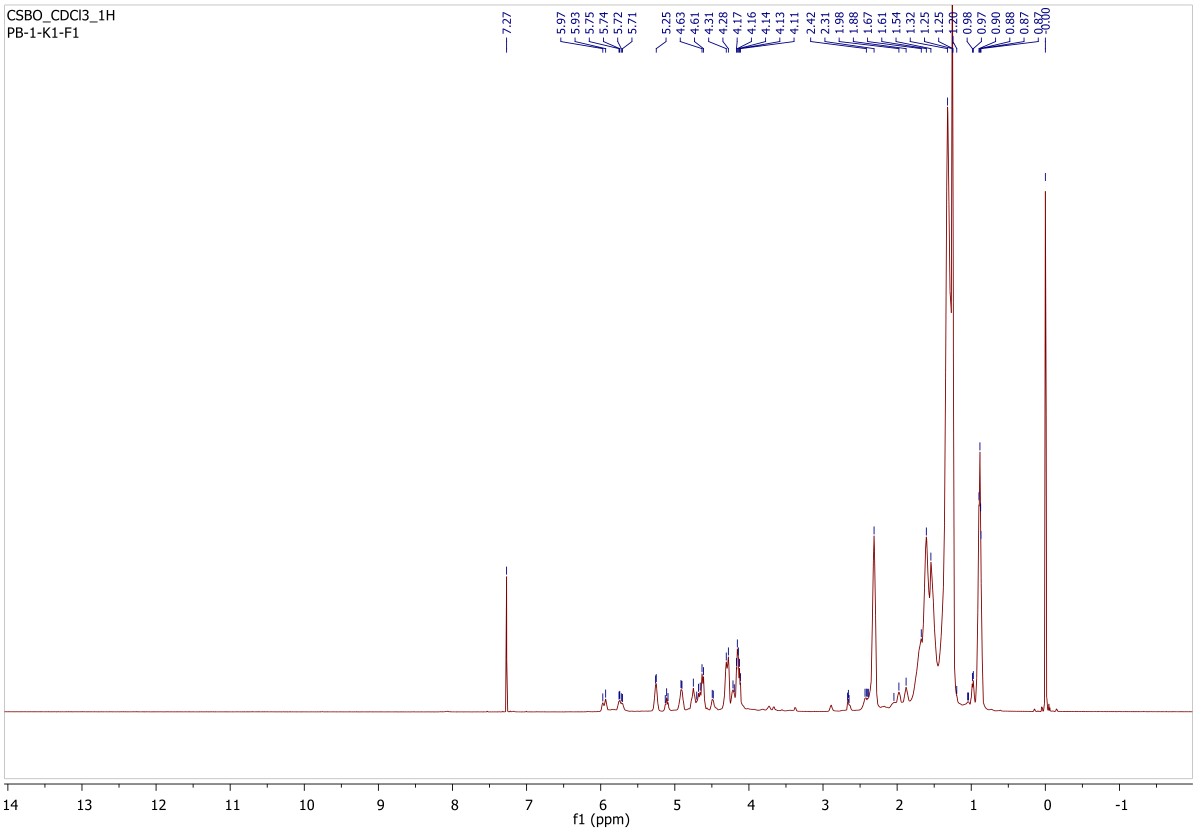

Fig. S3 ^{1}H NMR spectrum of [bmim]Ac.

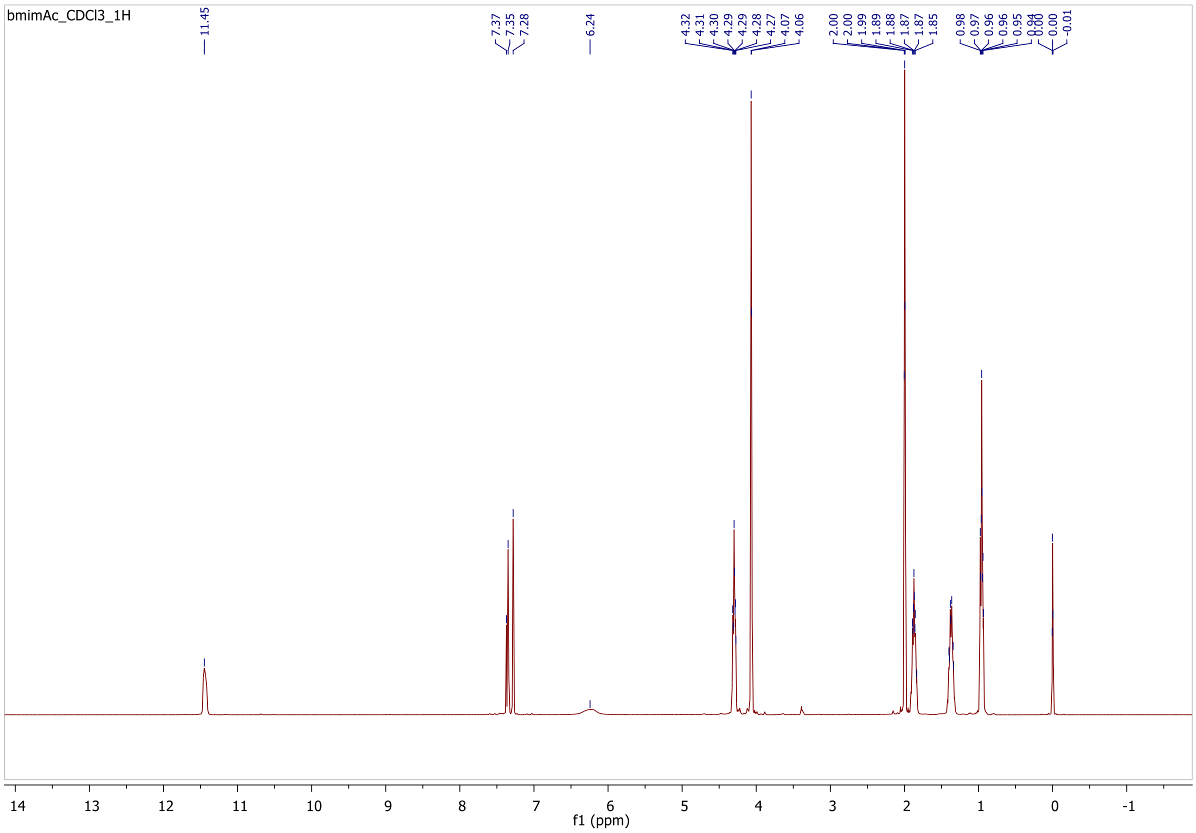

Fig. S4 ^{13}C NMR spectrum of [bmim]Ac.

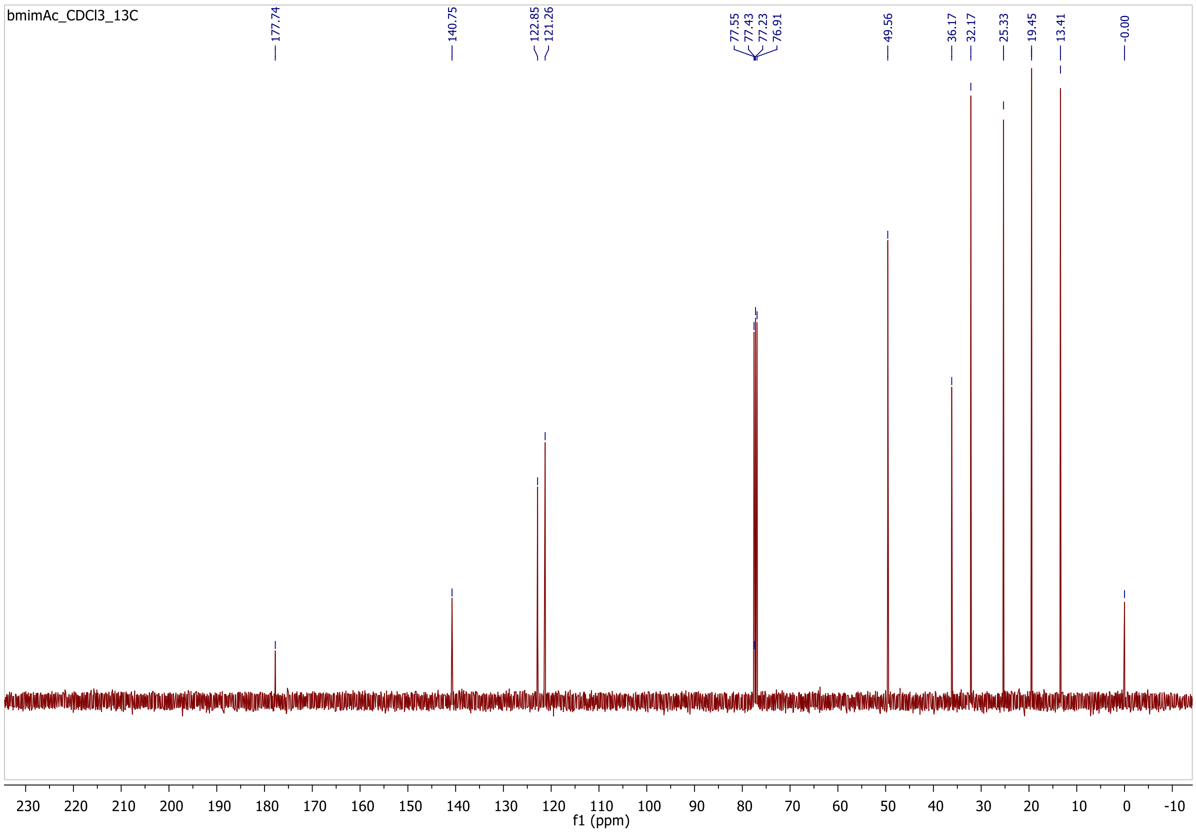

Fig. S5 ^{1}H NMR spectrum of [bmim]Br.

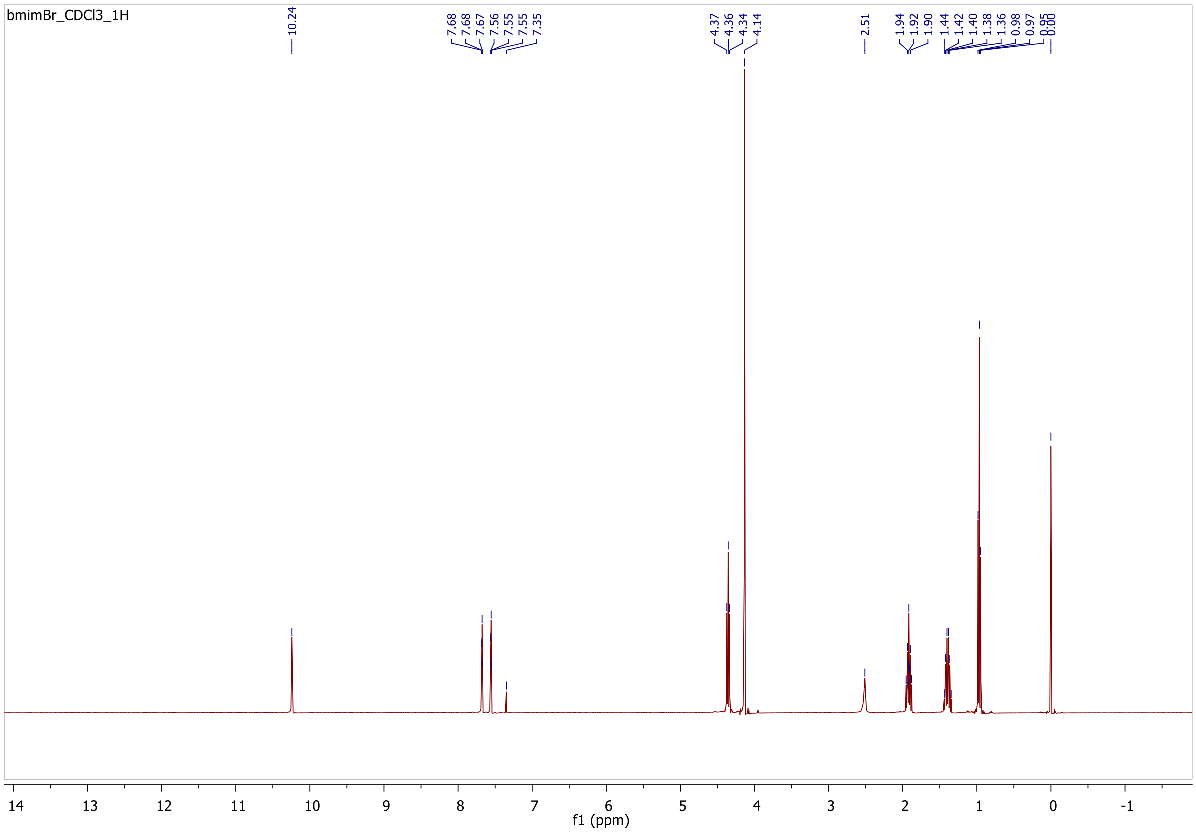

Fig. S6 ^{13}C NMR spectrum of [bmim]Br.

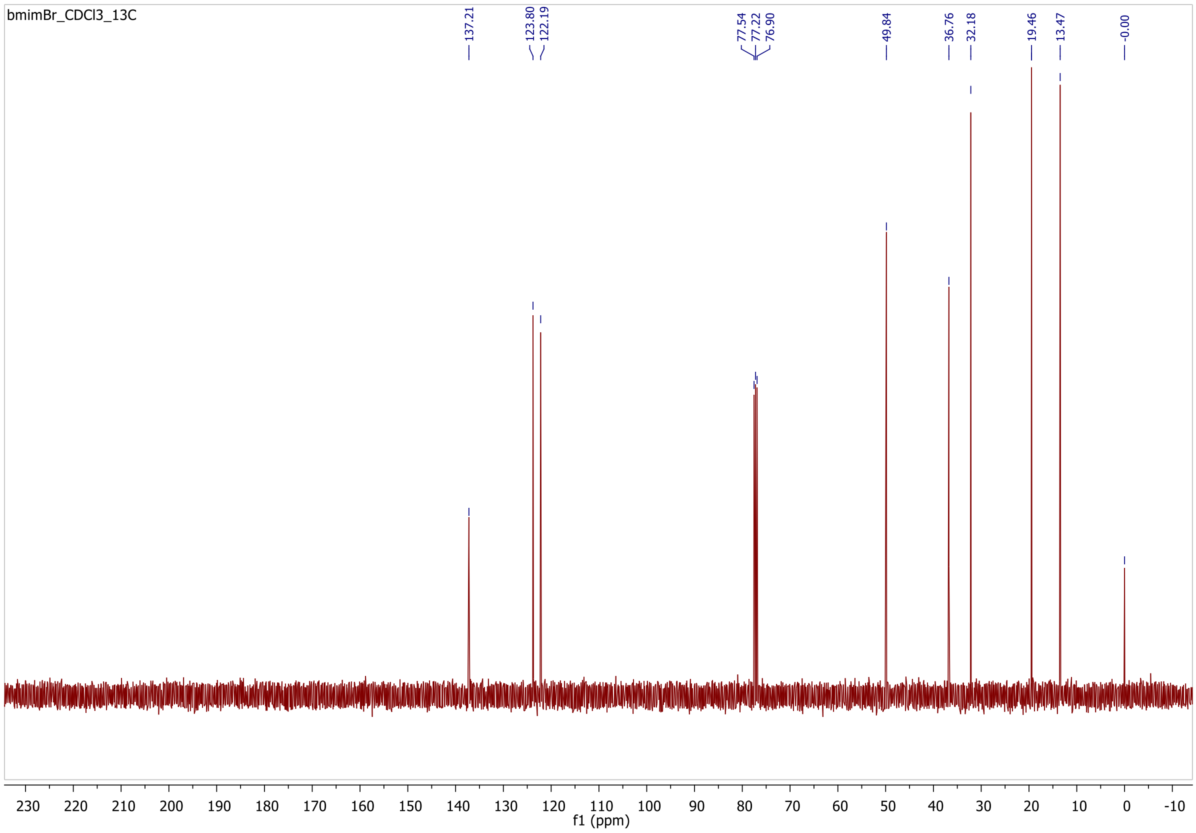

Fig. S7 ^{1}H NMR spectrum of [bmim]Cl.

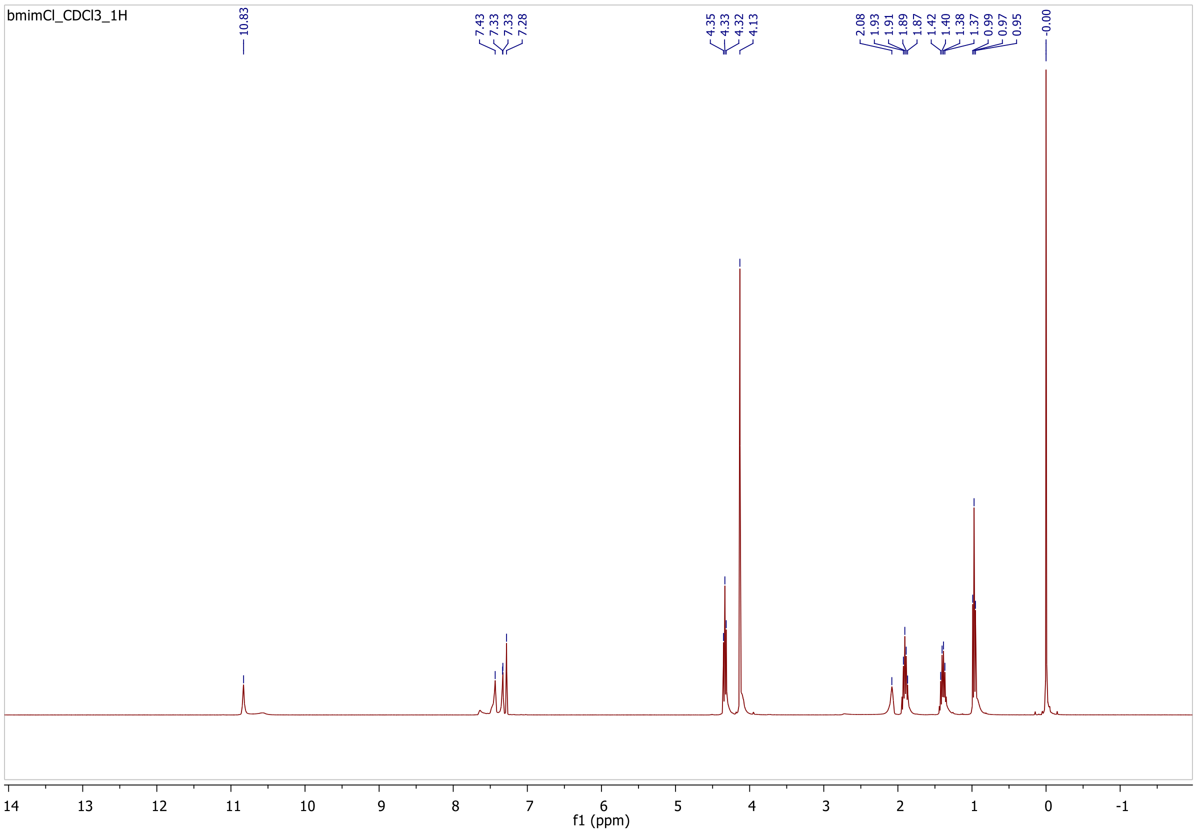

Fig. S8 ^{13}C NMR spectrum of [bmim]Cl.

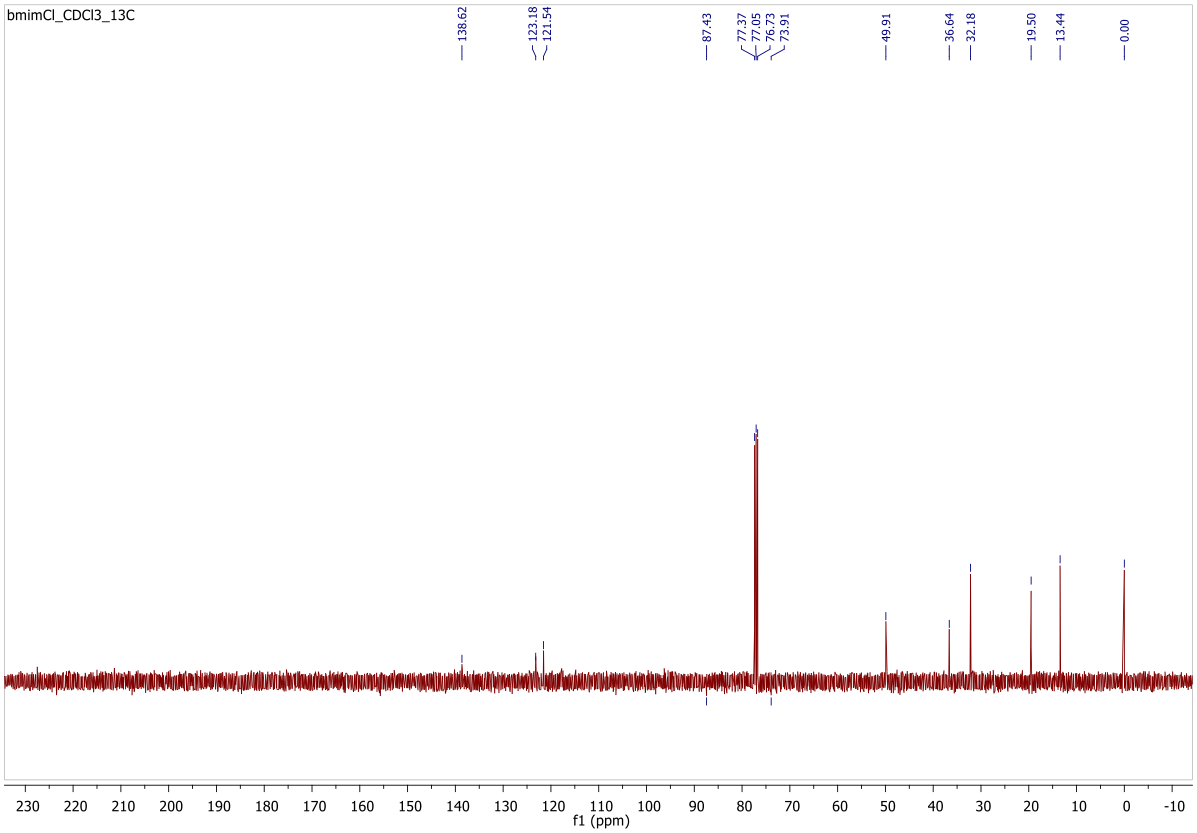

Fig. S9 ^{1}H NMR spectrum of [bmim]DMP.

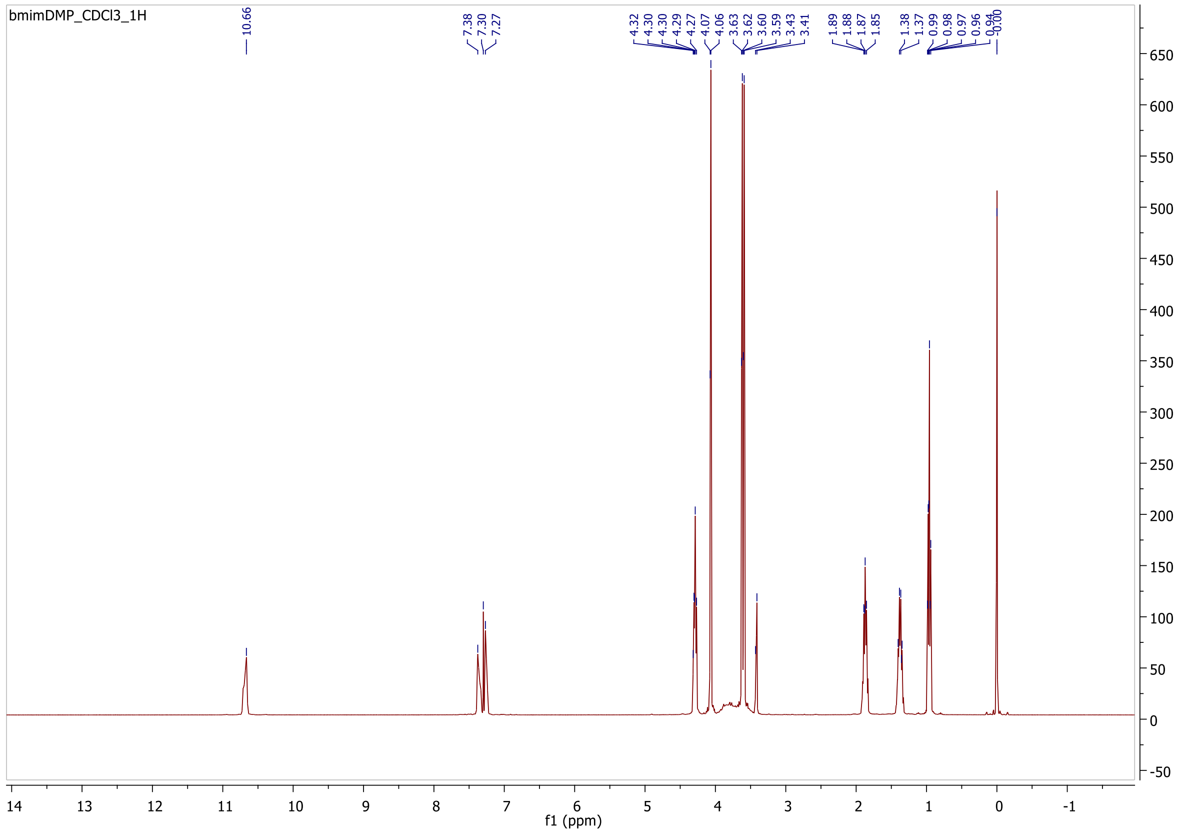

Fig. S10 ^{13}C NMR spectrum of [bmim]DMP.

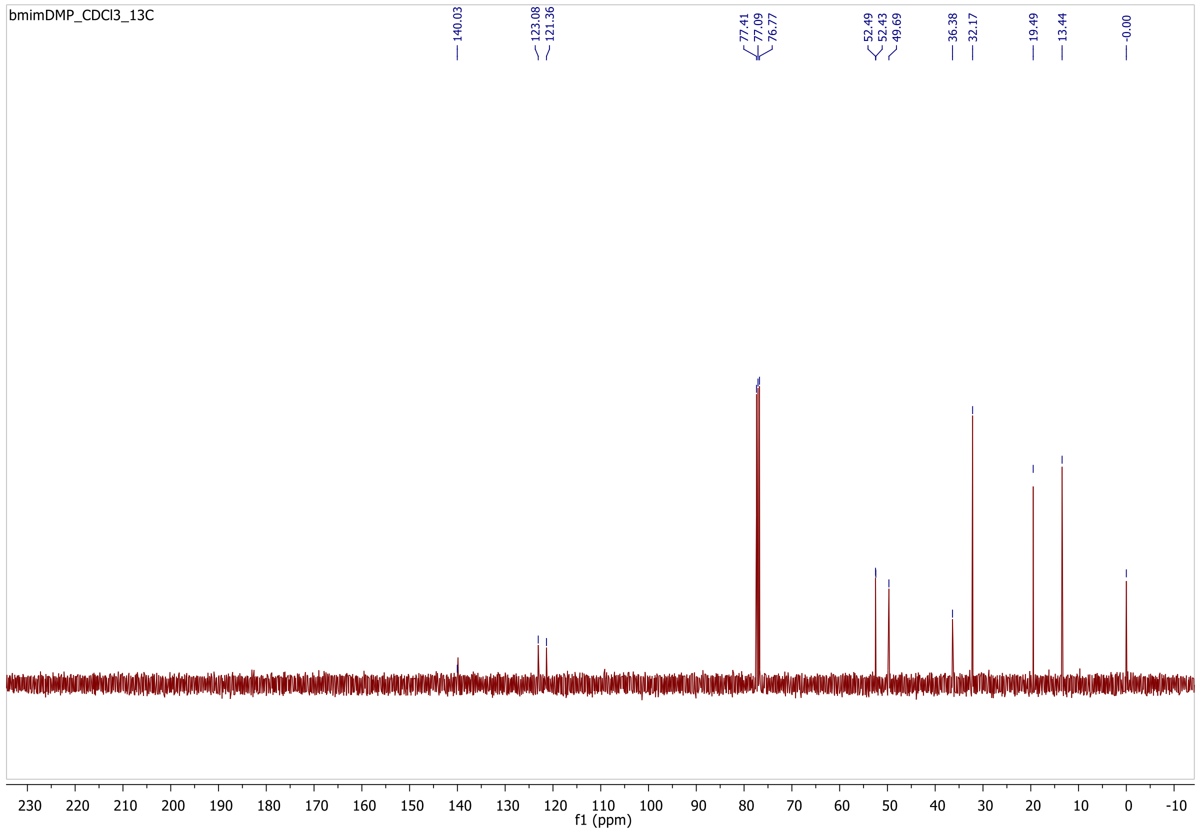

Fig. S11 ^{1}H NMR spectrum of $[bmim]N(CN)_2$.

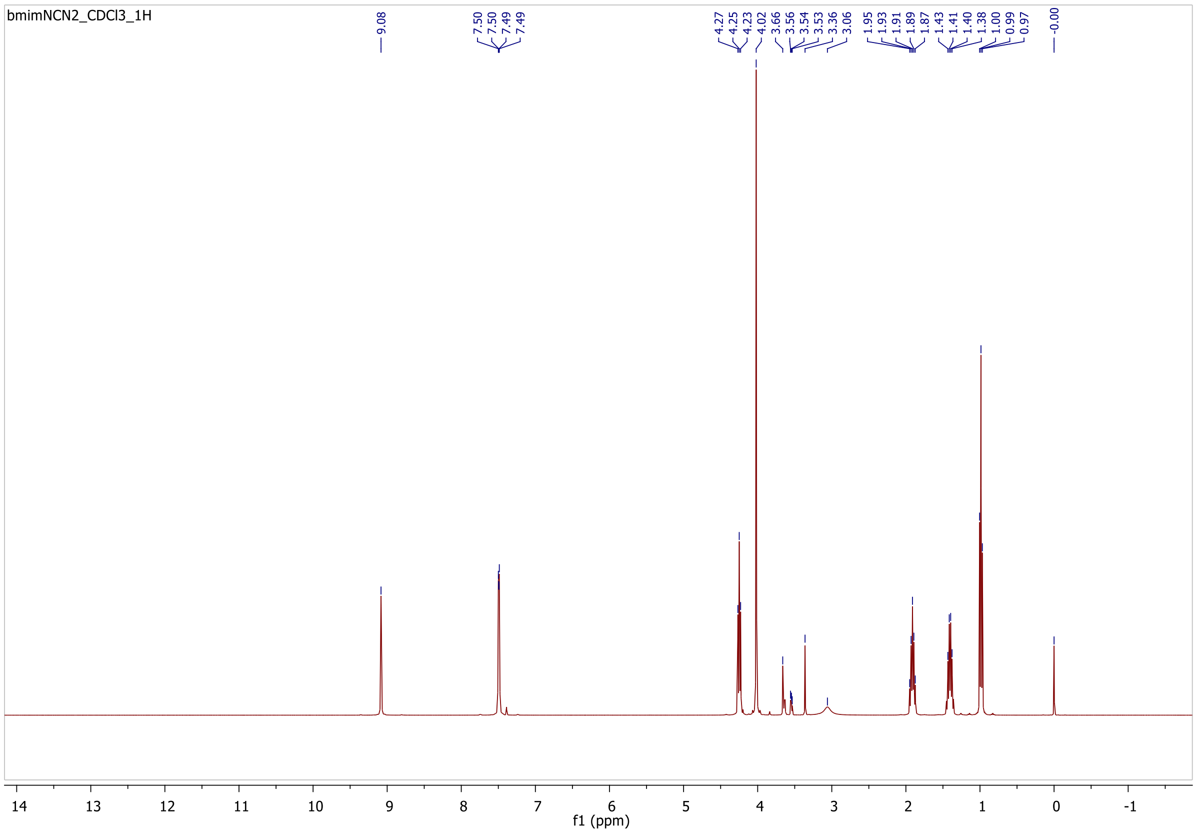

Fig. S12 ^{13}C NMR spectrum of $[bmim]N(CN)_2$.

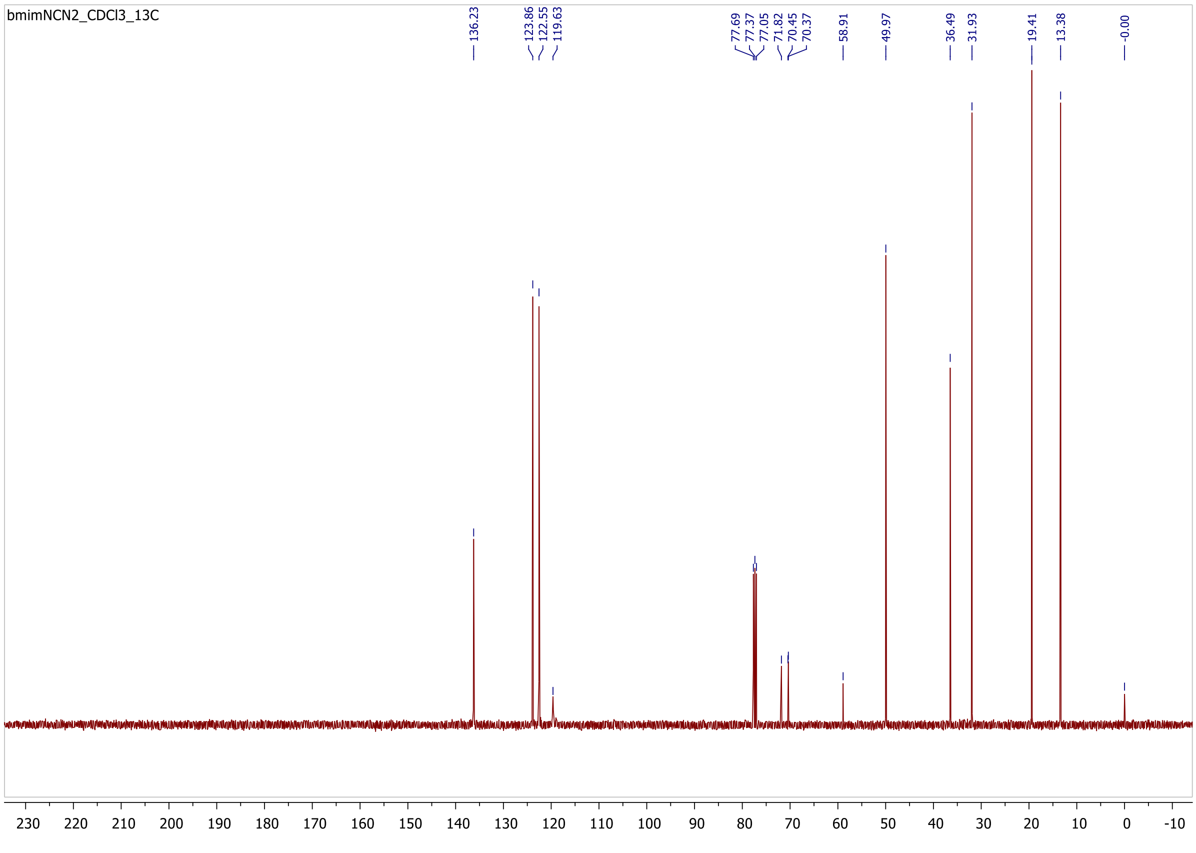

Fig. S13 ^{1}H NMR spectrum of [bmim]HSO_4.

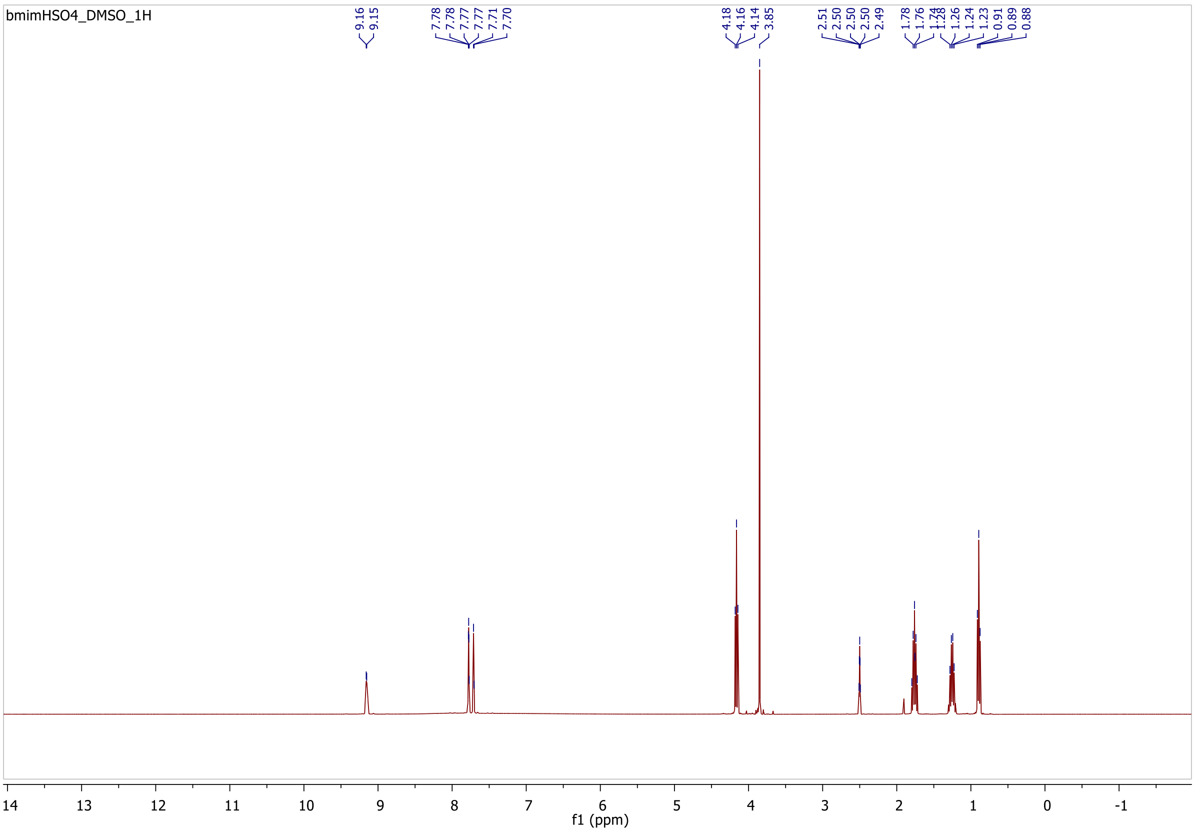

Fig. S14 ^{13}C NMR spectrum of [bmim]HSO_4.

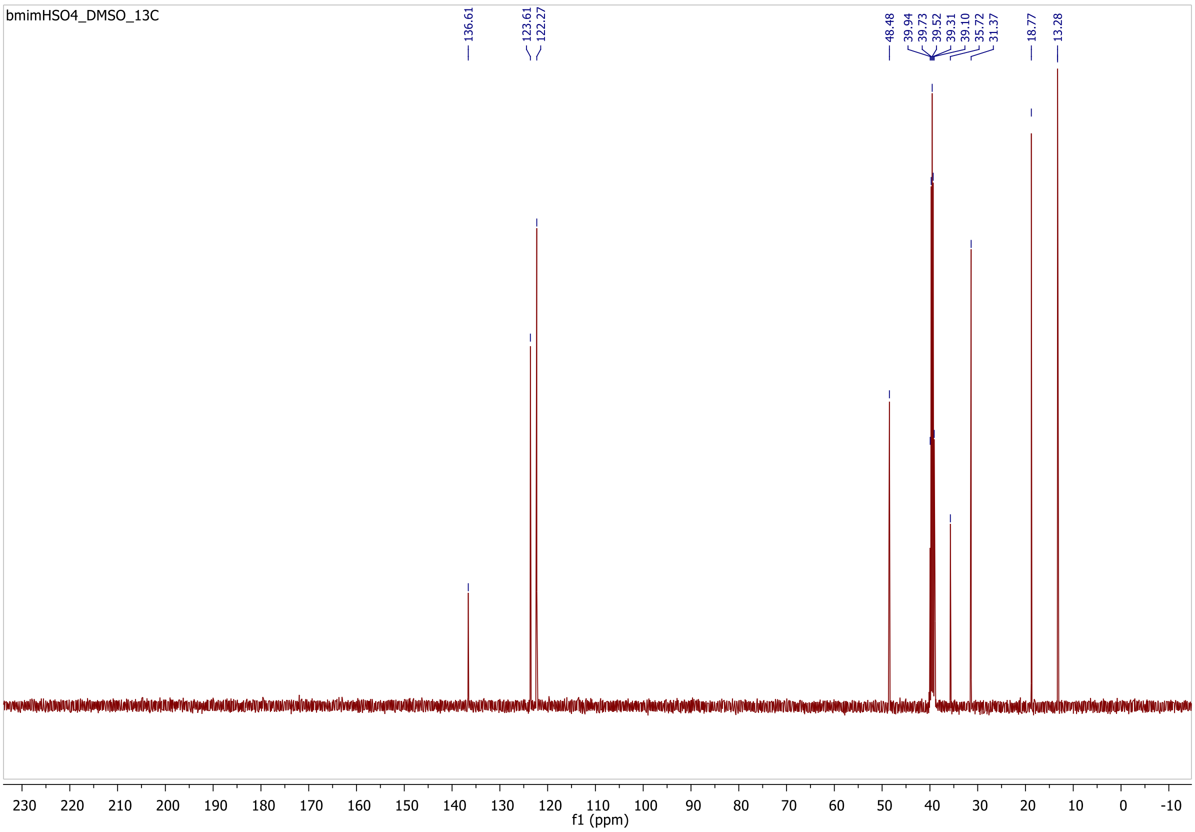

Fig. S15 ^{1}H NMR spectrum of ChAc.

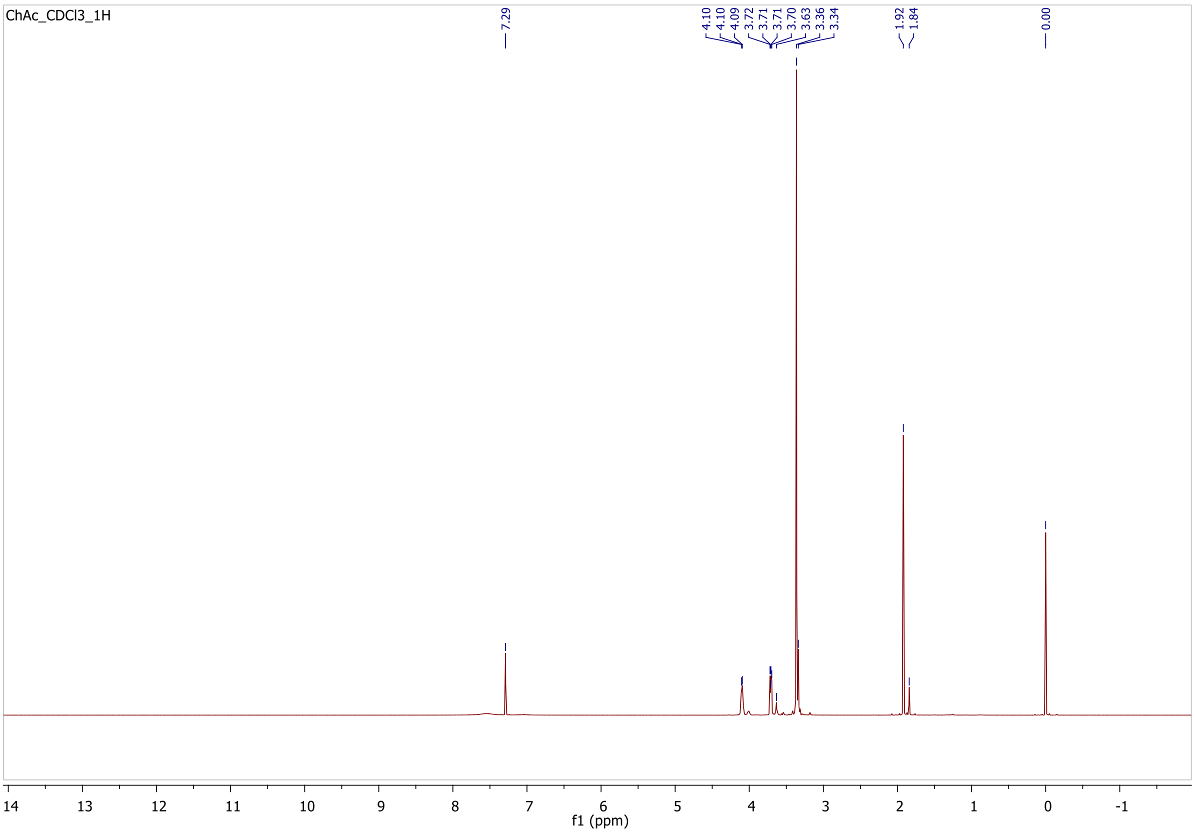

Fig. S16 ^{13}C NMR spectrum of ChAc.

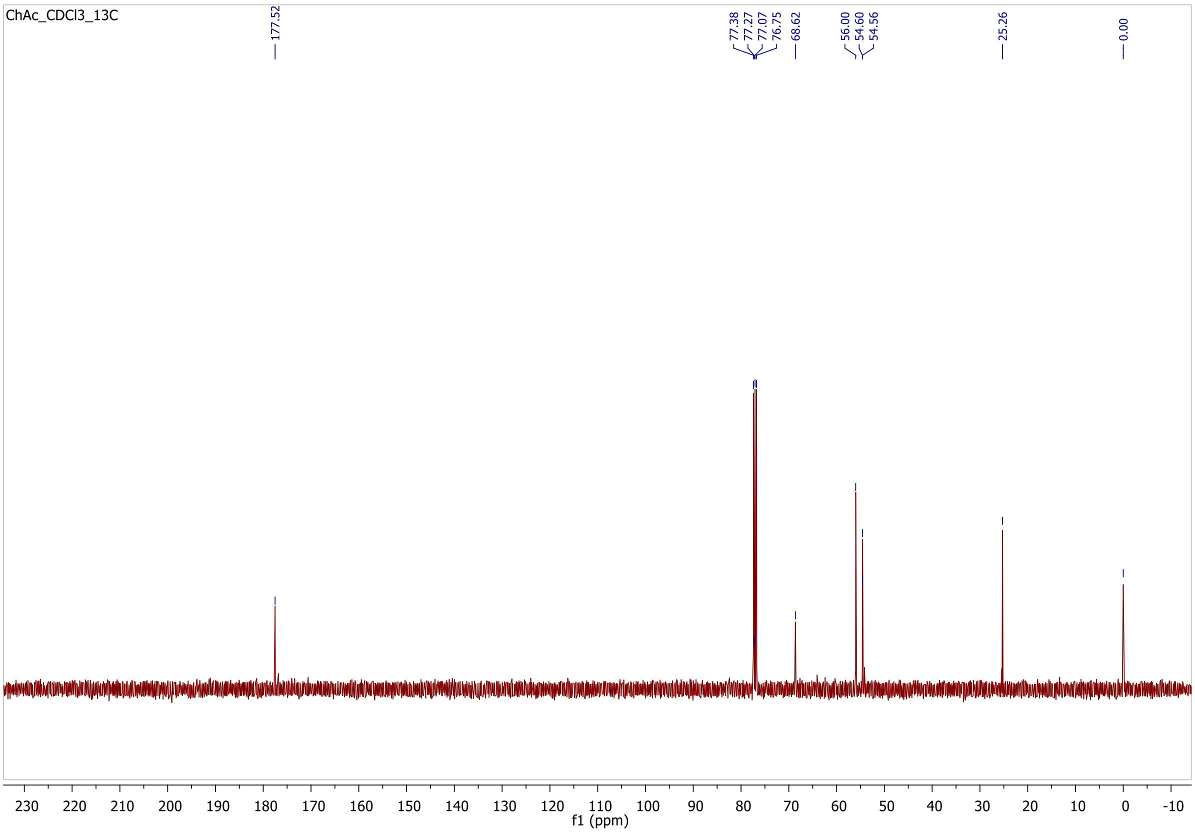

Fig. S17 ^{1}H NMR spectrum of [emim]Br.

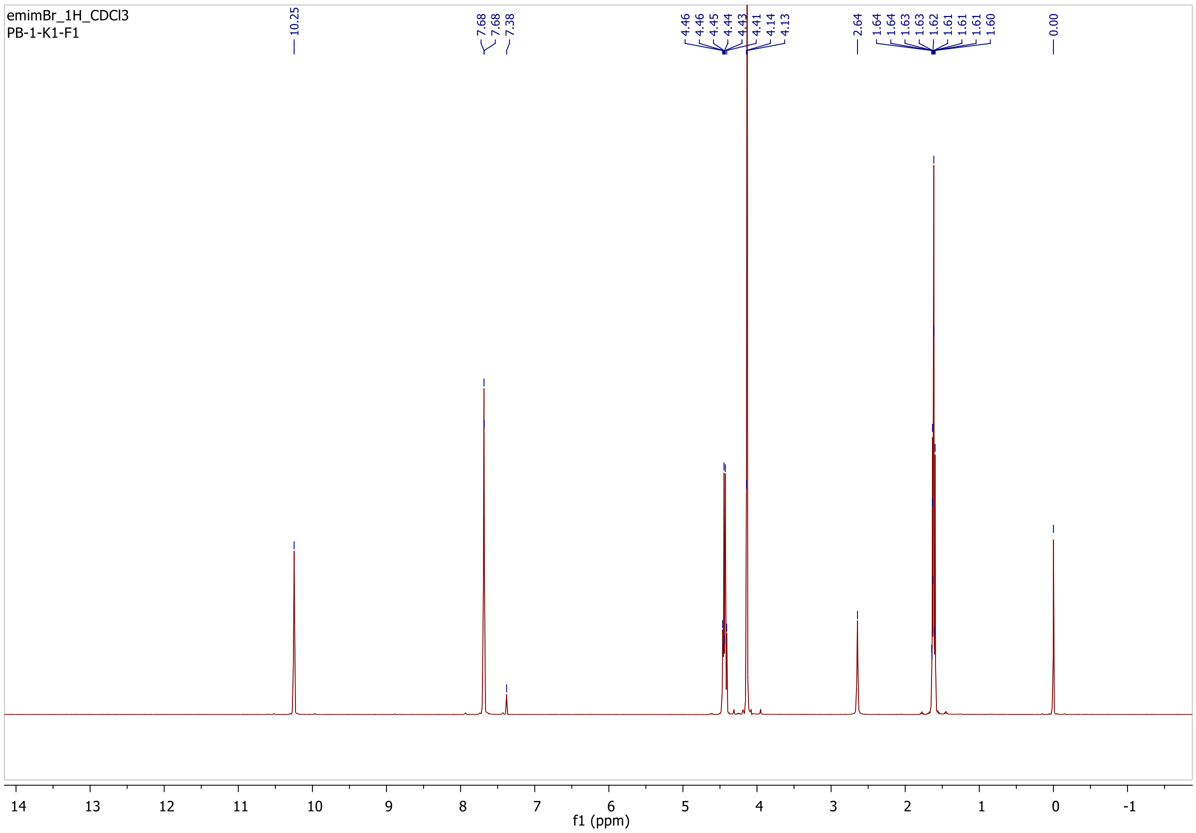

Fig. S18 ^{13}C NMR spectrum of [emim]Br.

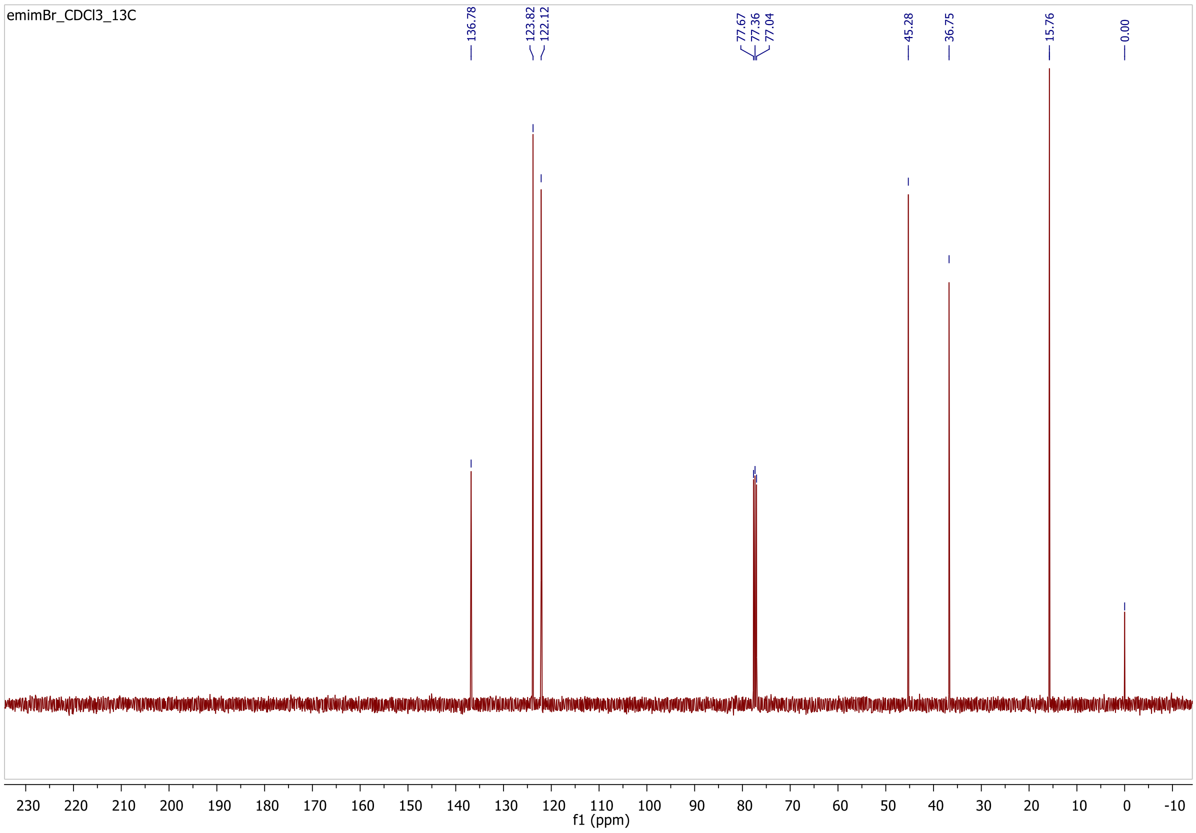

Fig. S19 ^{1}H NMR spectrum of [emim]Cl.

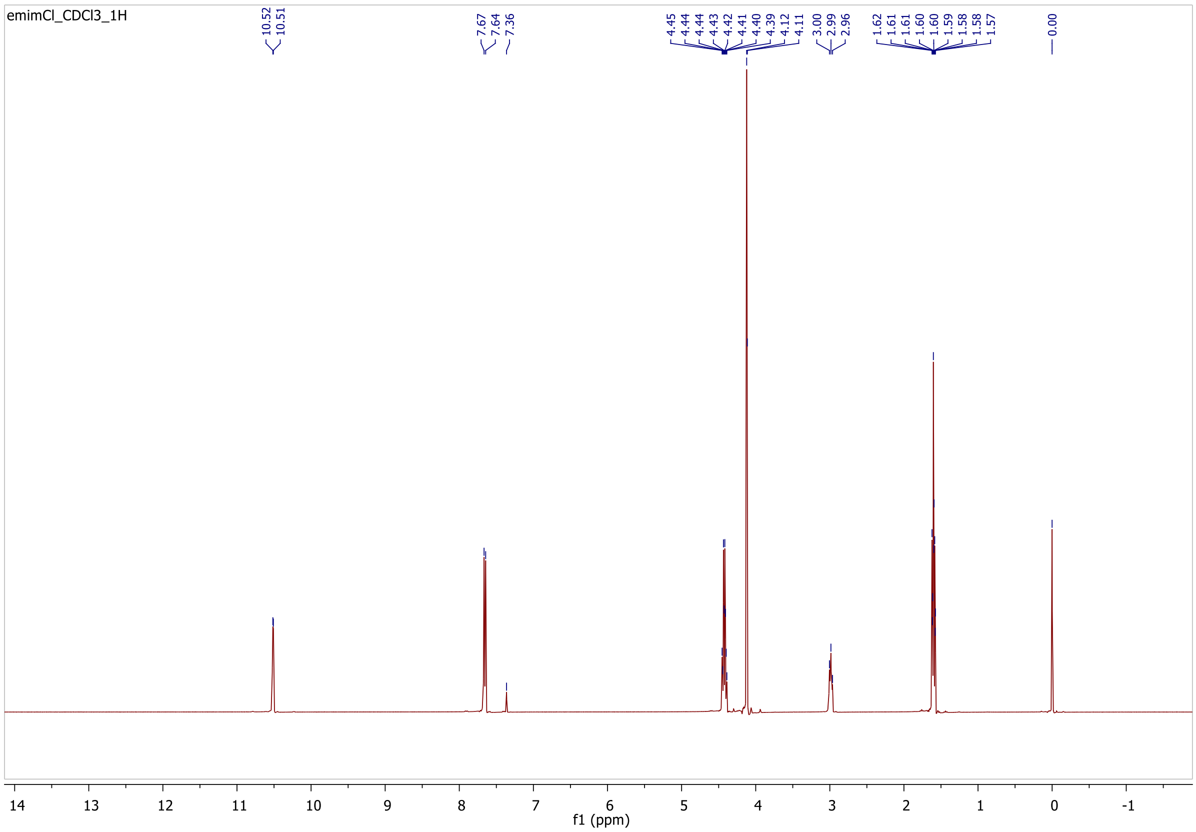

Fig. S20 ^{13}C NMR spectrum of [emim]Cl.

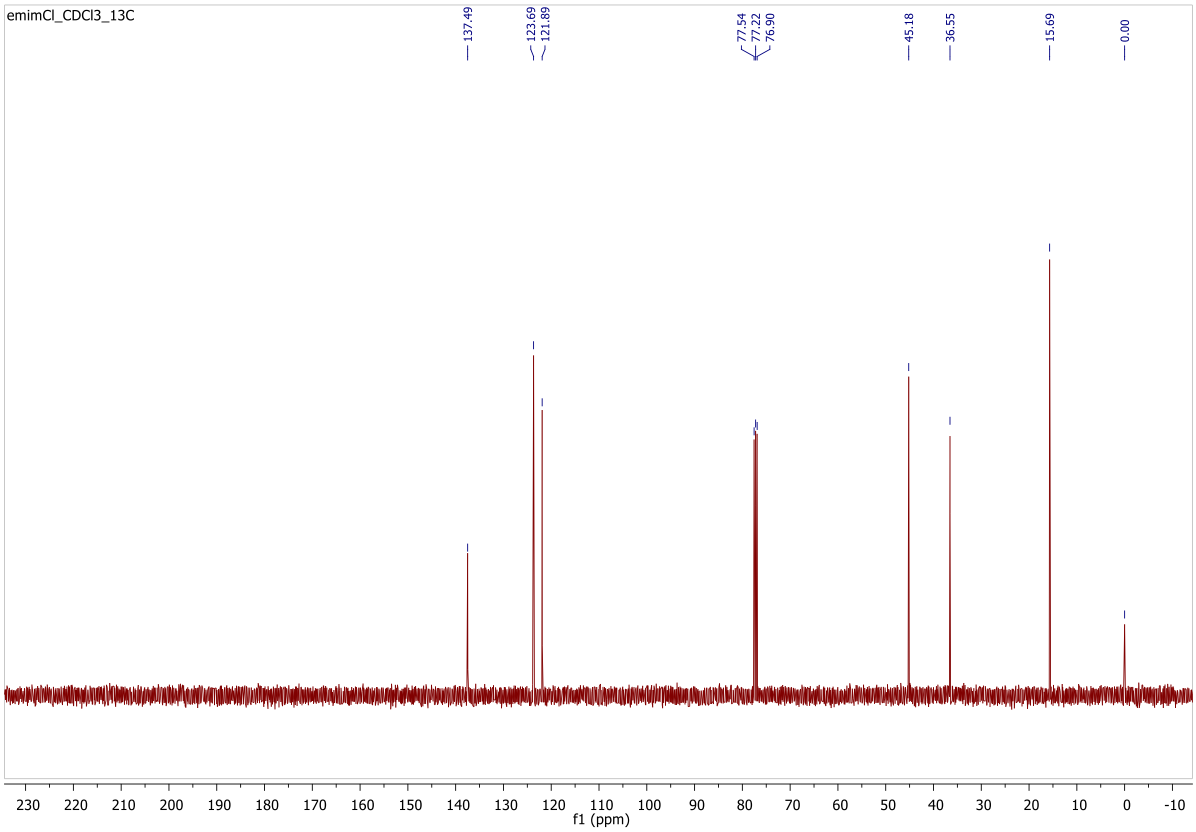

Fig. S21 ^{1}H NMR spectrum of [emim]DBP.

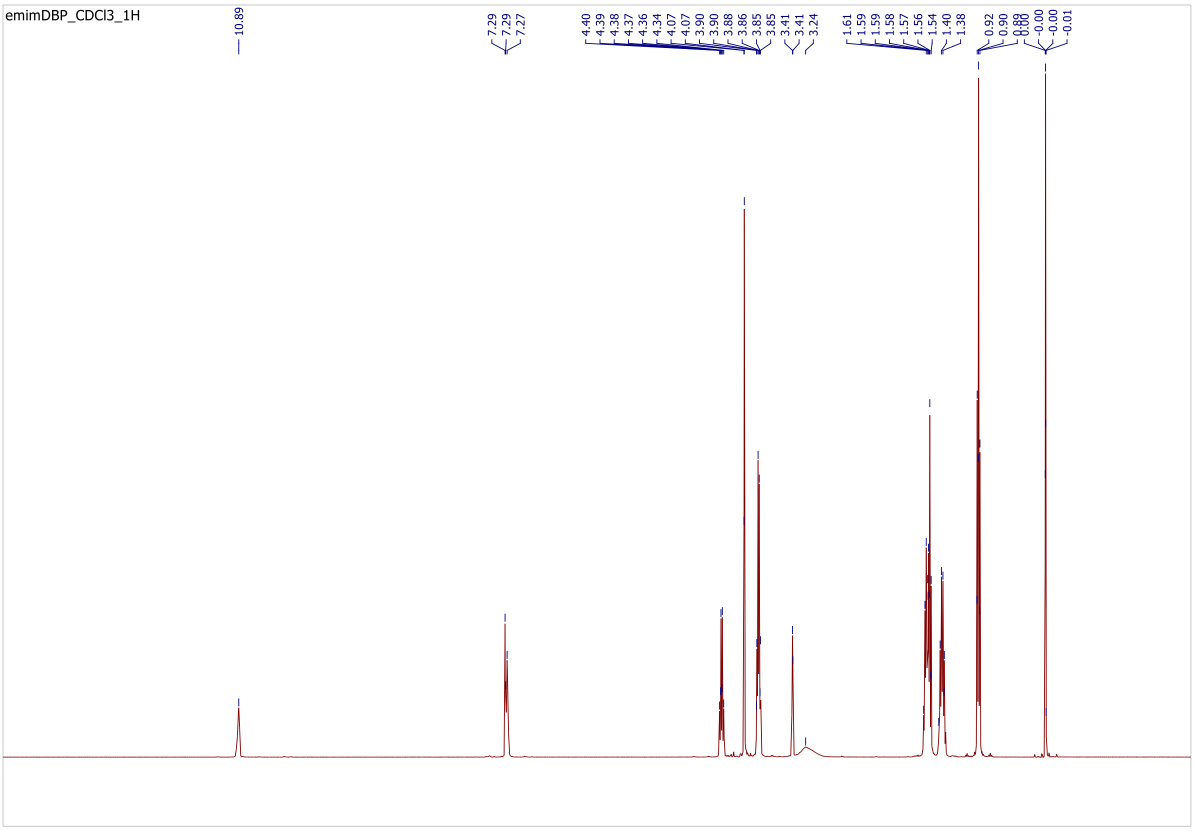

Fig. S22 ^{13}C NMR spectrum of [emim]DBP.

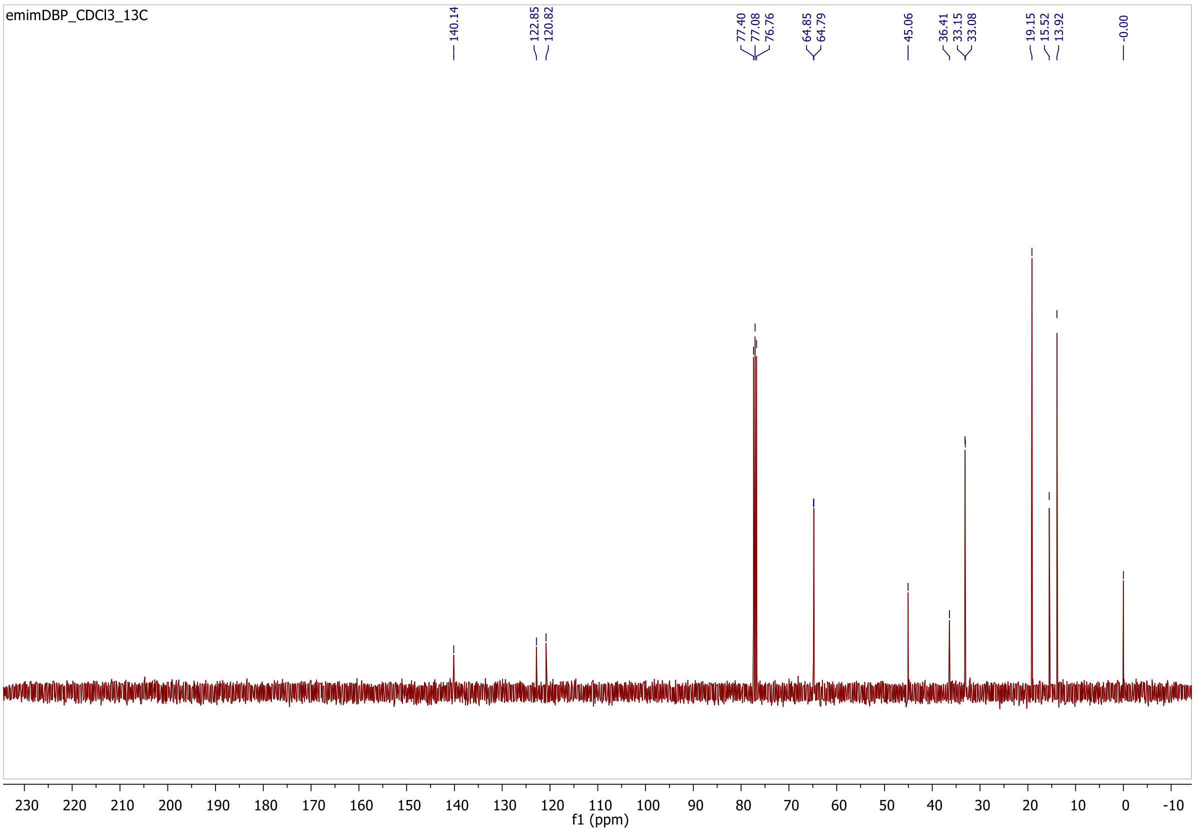

Fig. S23 ^{1}H NMR spectrum of [emim]$EtSO_4$.

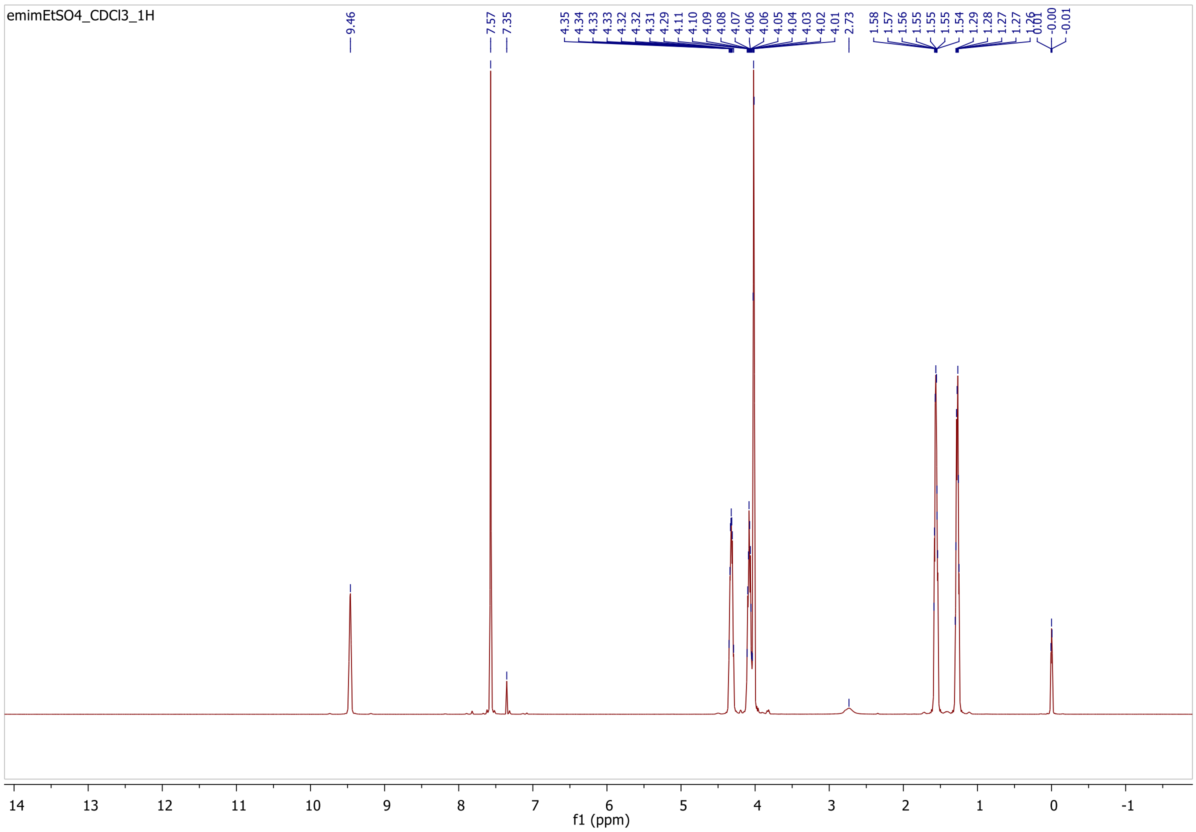

Fig. S24 ^{13}C NMR spectrum of [emim]$EtSO_4$.

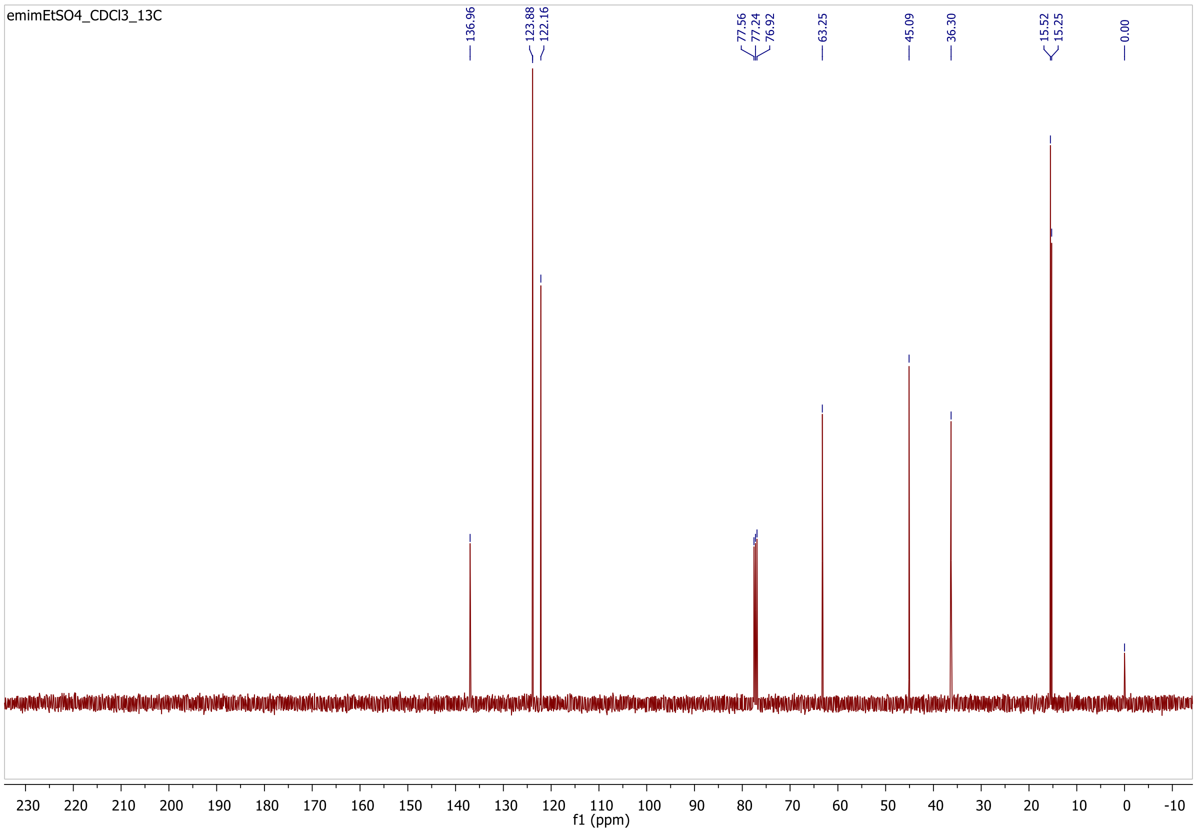

Fig. S25 ^{1}H NMR spectrum of [emim]HSO_4.

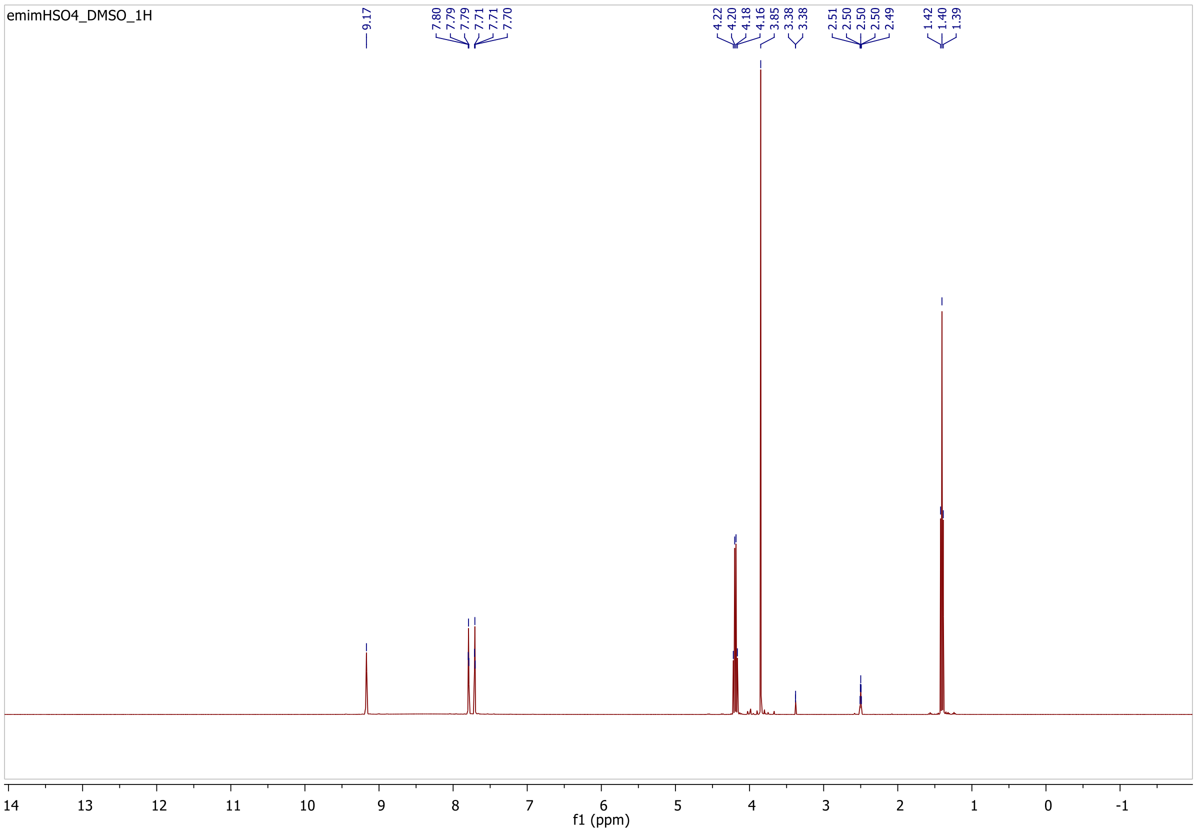

Fig. S26 ^{13}C NMR spectrum of [emim]HSO_4.

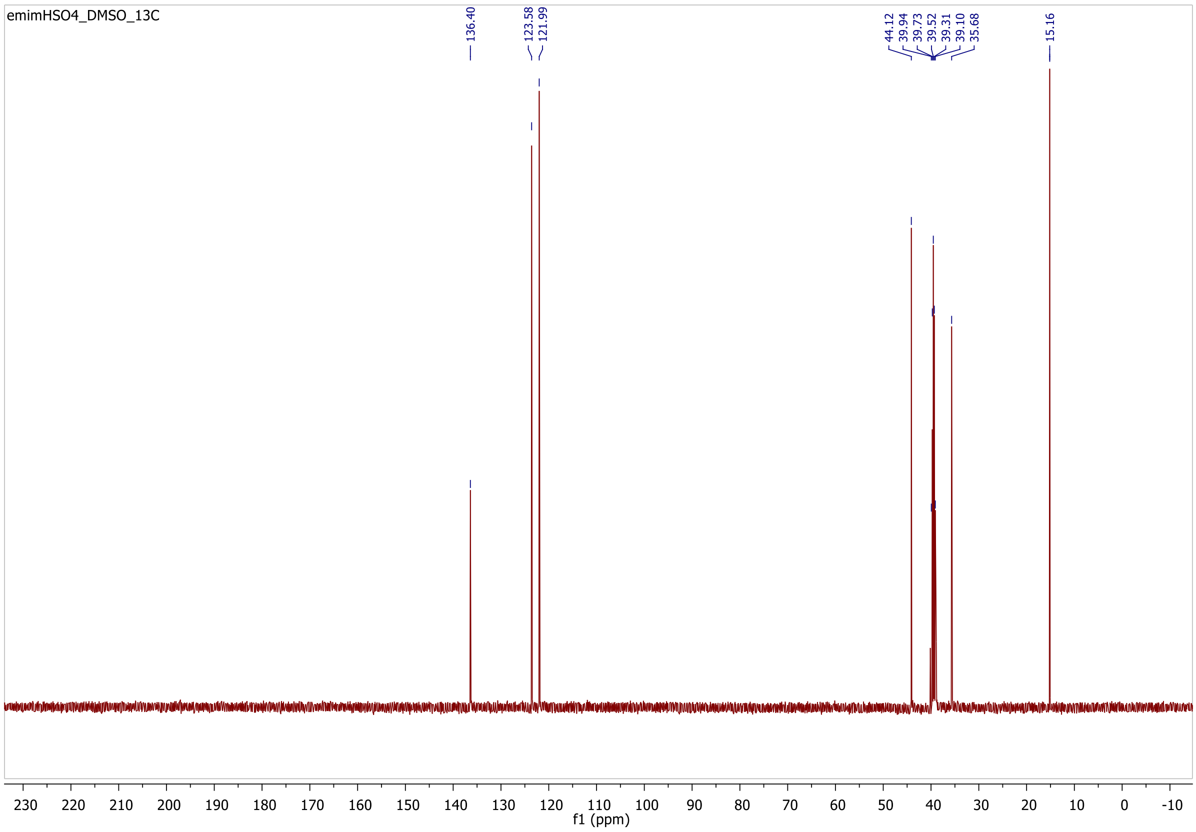

Fig. S27 ^{1}H NMR spectrum of [hmim]Br.

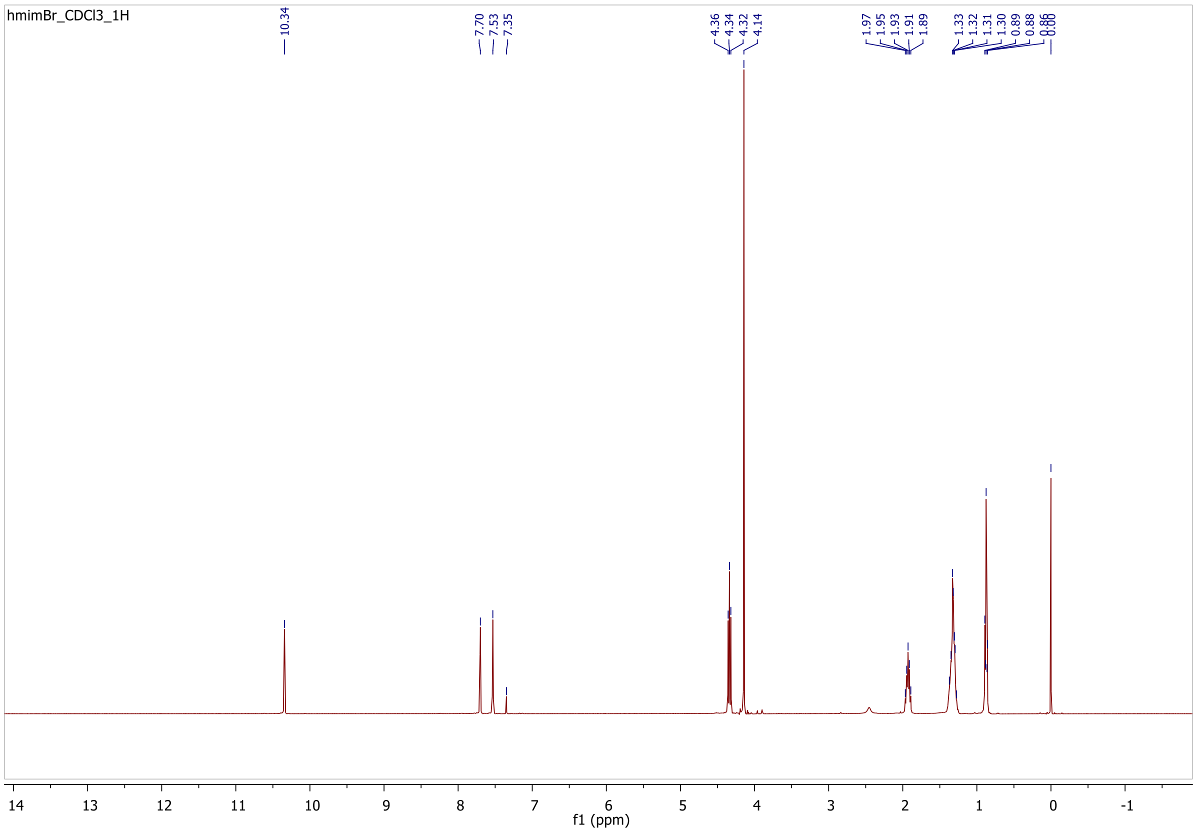

Fig. S28 ^{13}C NMR spectrum of [hmim]Br.

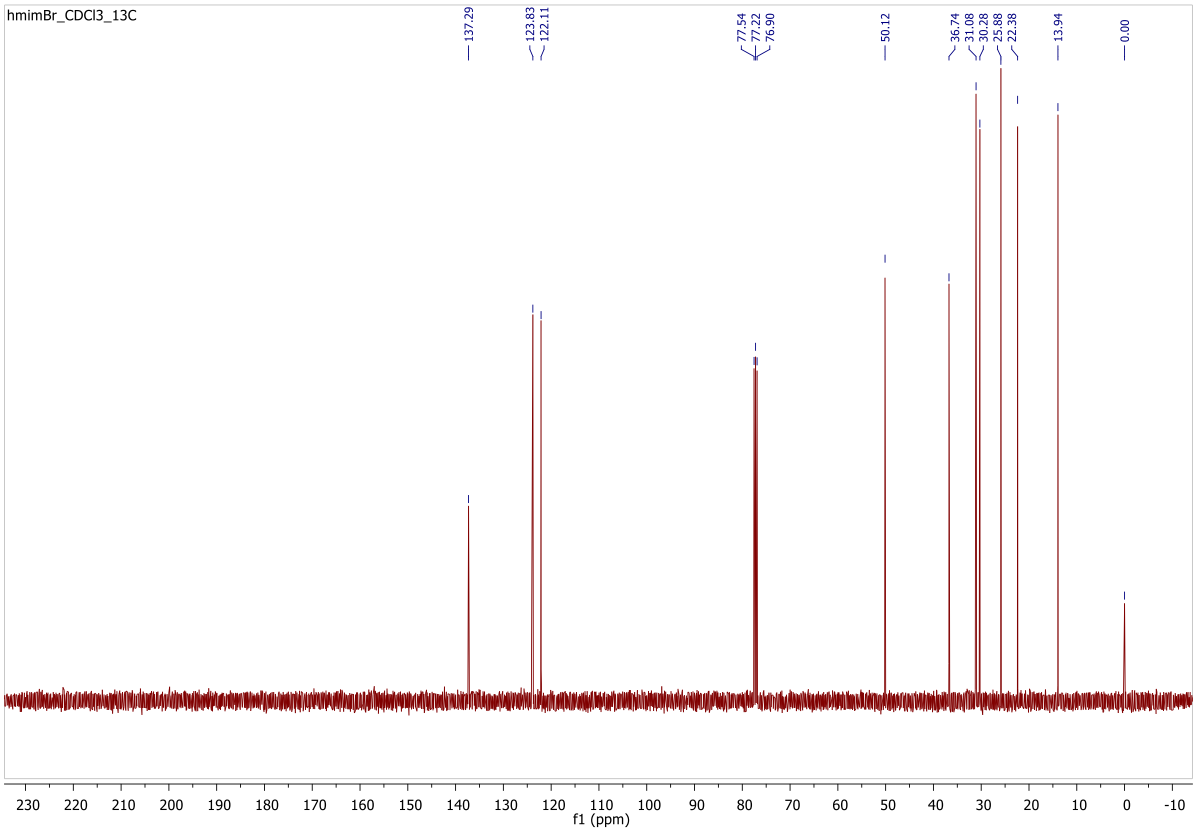

Fig. S29 ^{1}H NMR spectrum of [hmim]Cl

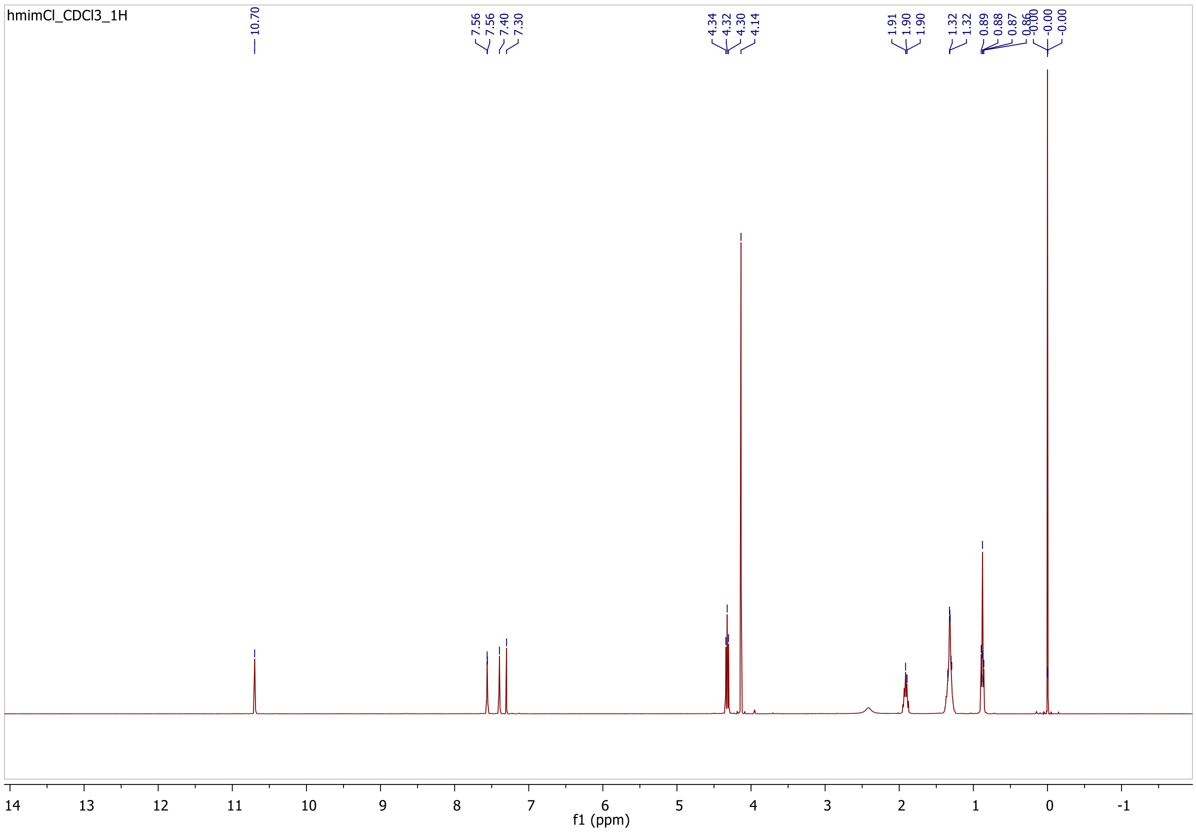

Fig. S30 ^{13}C NMR spectrum of [hmim]Cl.

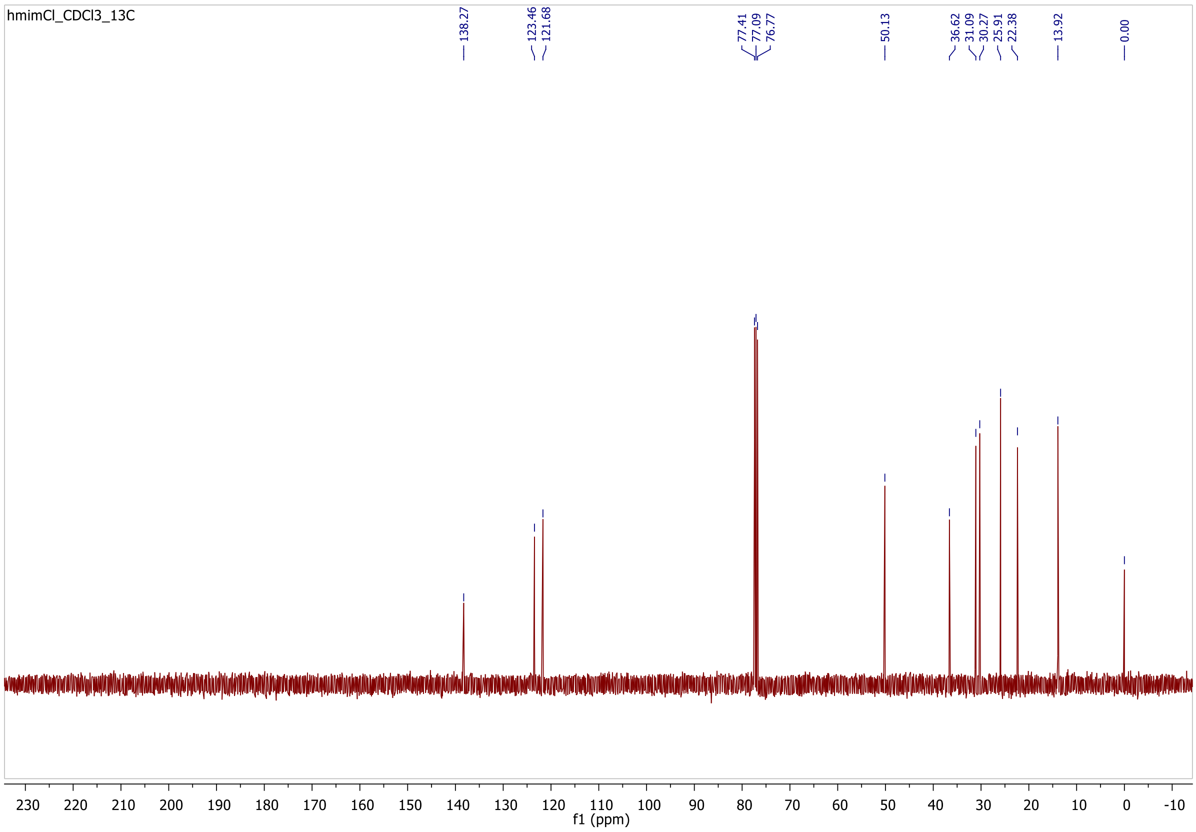

Fig. S31 ^{1}H NMR spectrum of [ocmim]Cl.

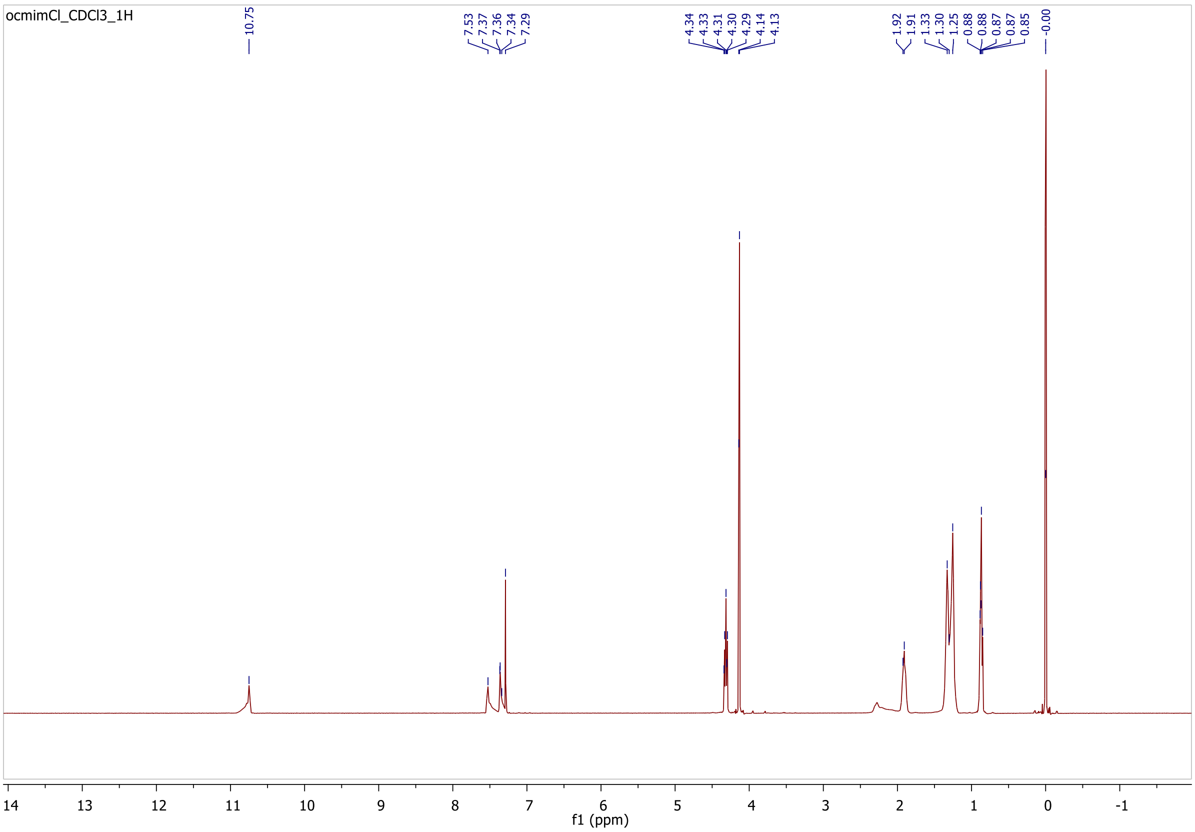

Fig. S32 ^{13}C NMR spectrum of [omim]Cl.

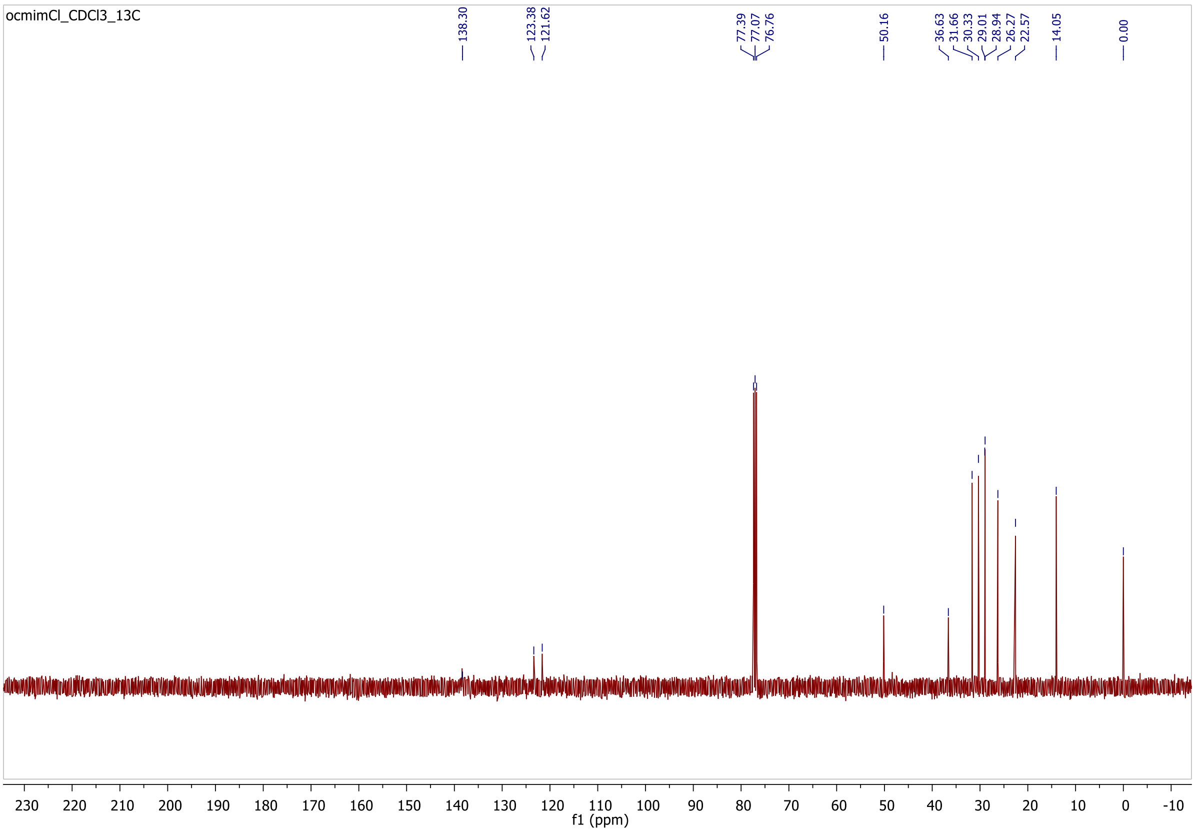

Fig. S33 ^{1}H NMR spectrum of TBABr.

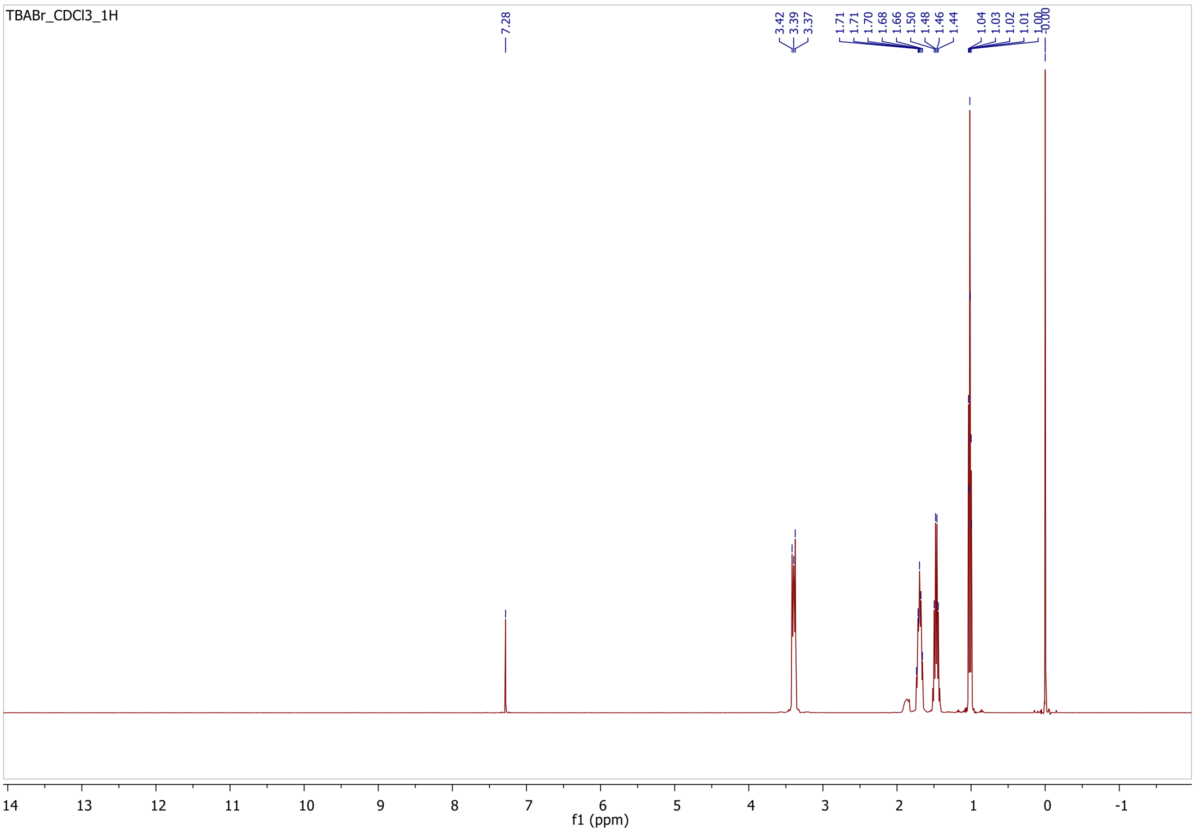

Fig. S34 ^{13}C NMR spectrum of TBABr.

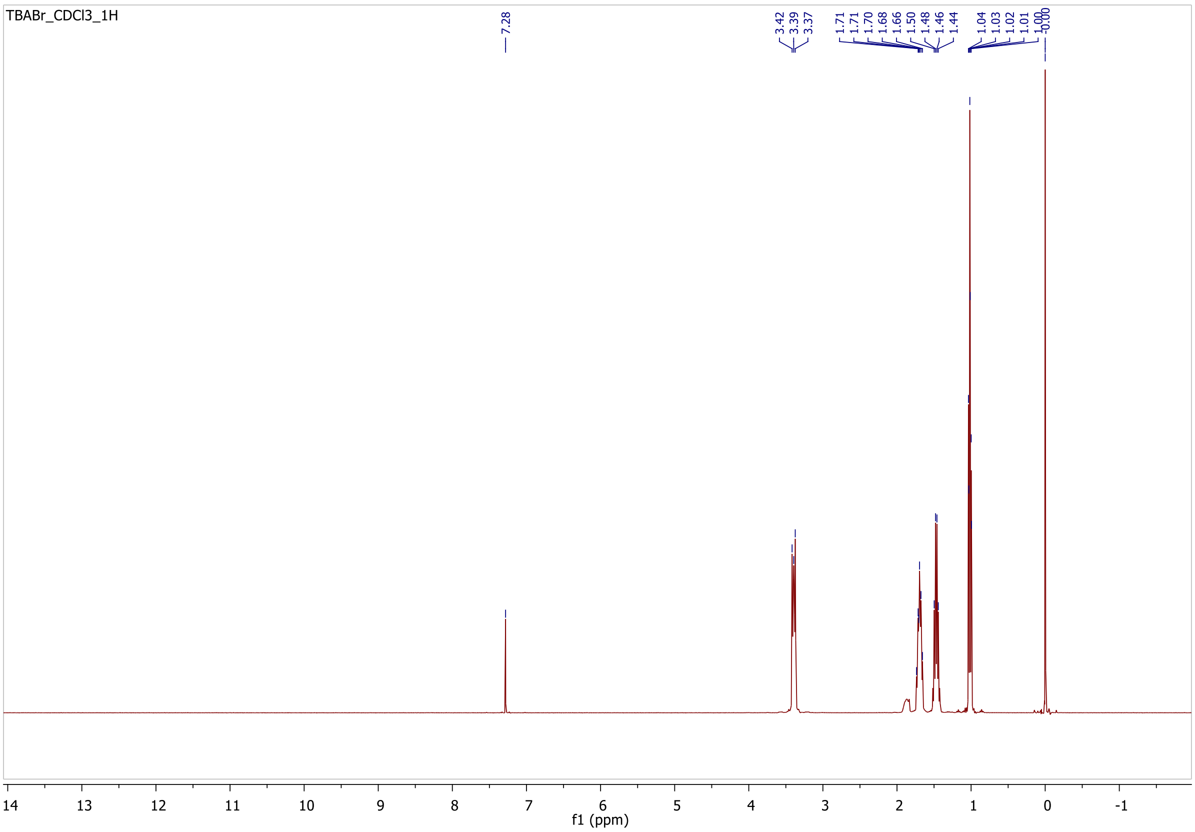

Fig. S35 ESI-MS spectrum of ESBO.

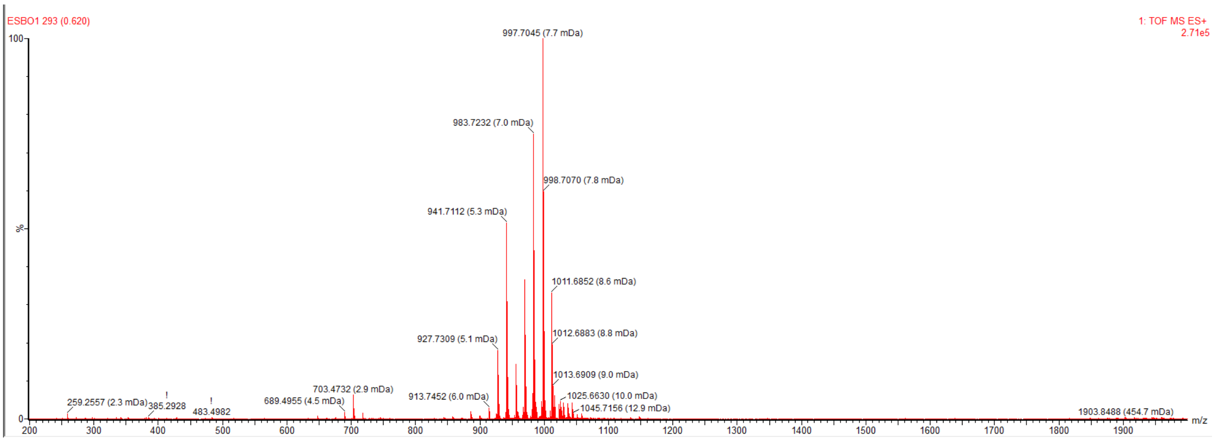

.

Fig. S36 ESI-MS spectrum of CC-[emim]Br.

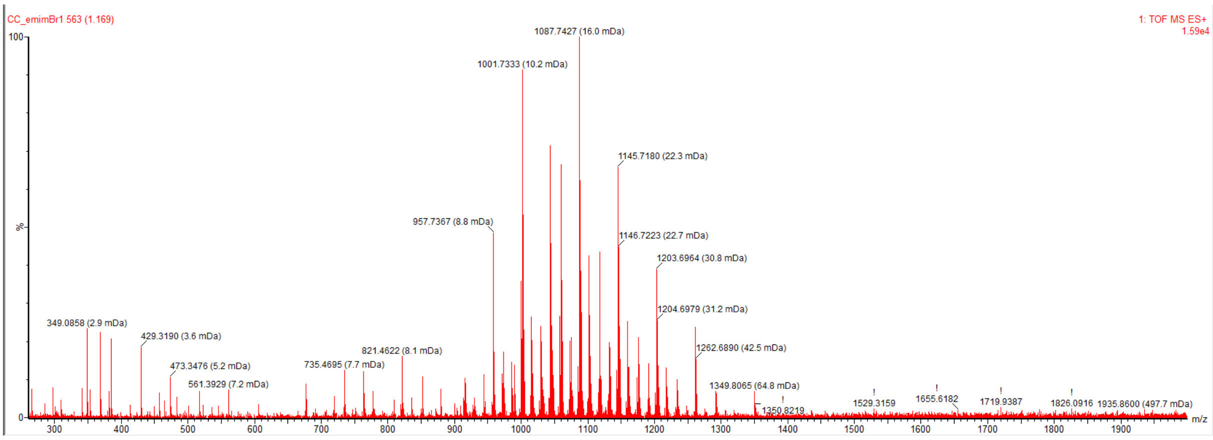

Fig. S37 ESI-MS spectrum of CC-TBABr.

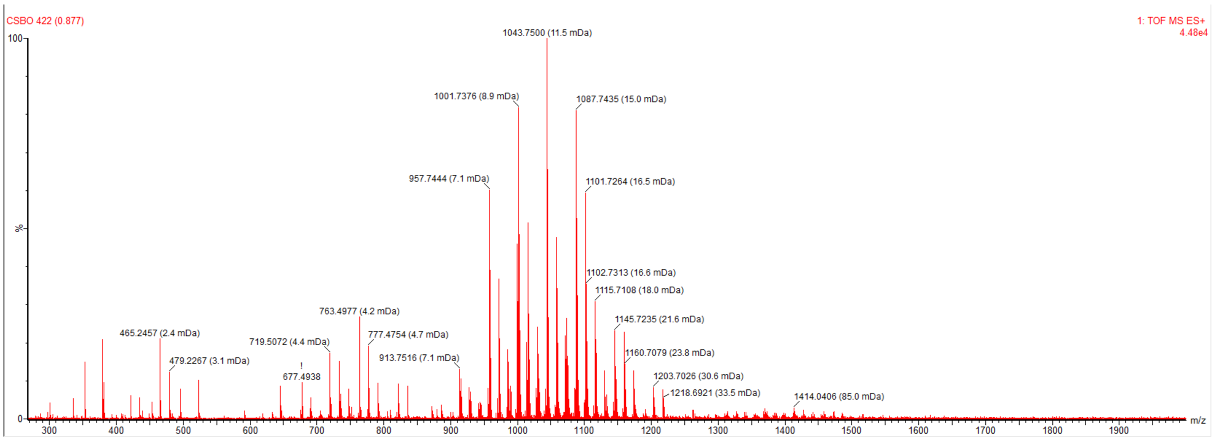

Fig. S38 TGA curves of TBAB-NIPU1 and IL-NIPU1.

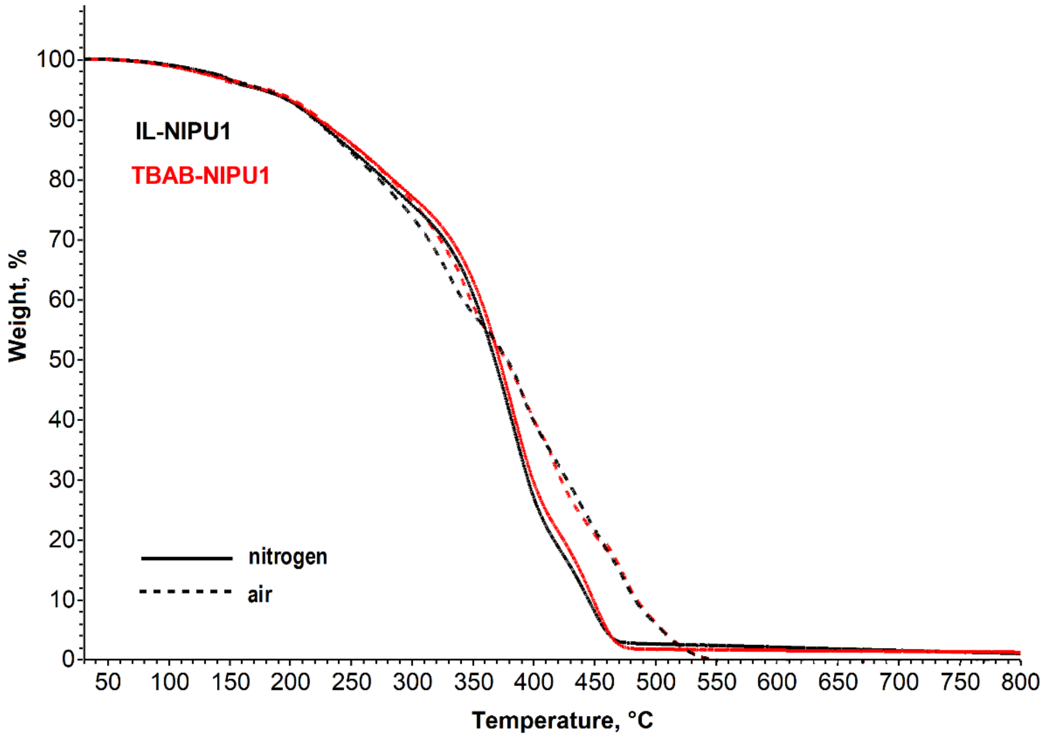

Fig. S39 DSC curves of TBAB-NIPU1 and IL-NIPU1.

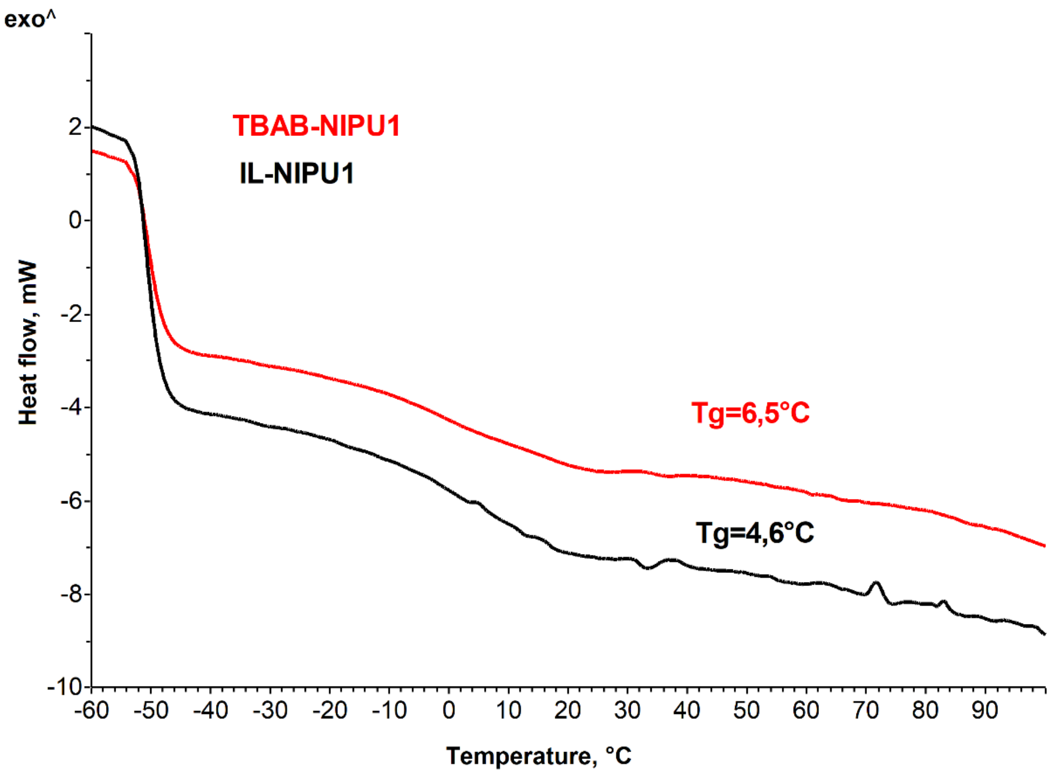

Fig. S40 TGA and DTG curves of NIPU foam.

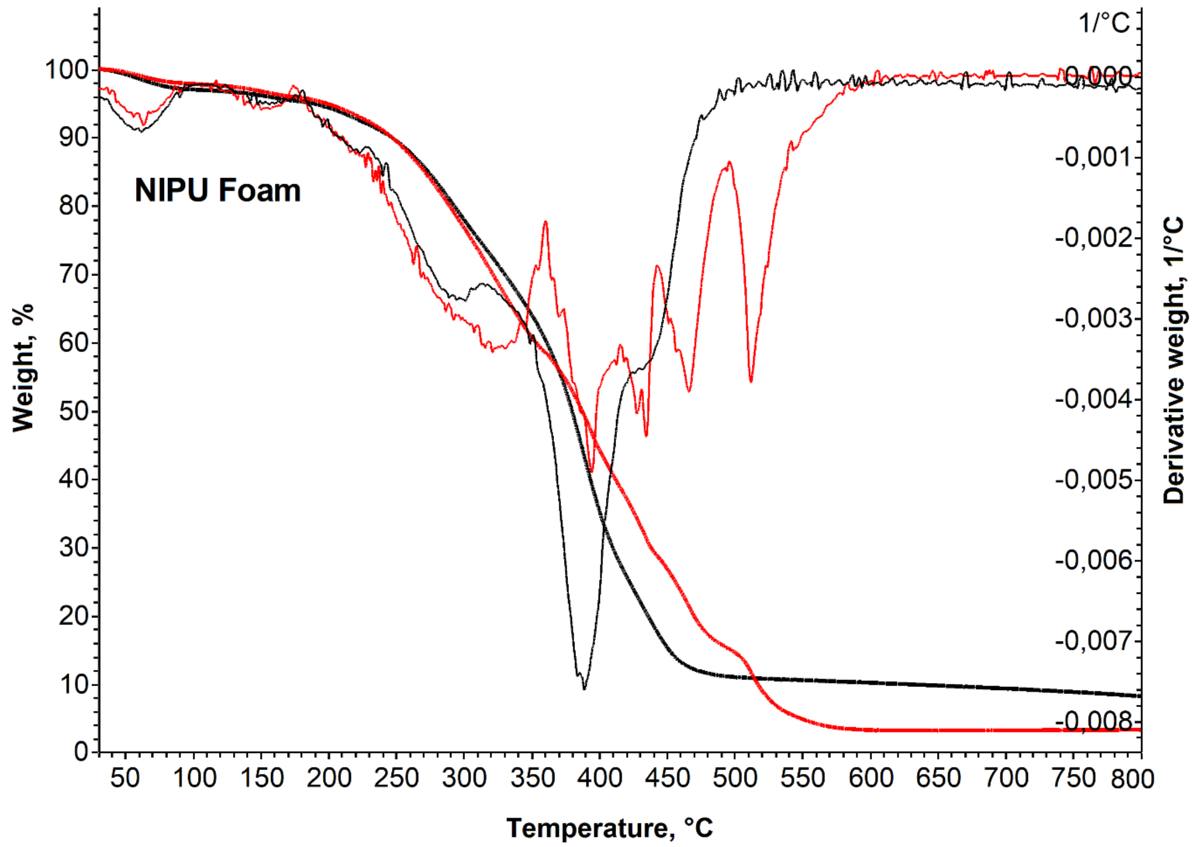

Fig. S41 FT-IR spectrum of DETA.

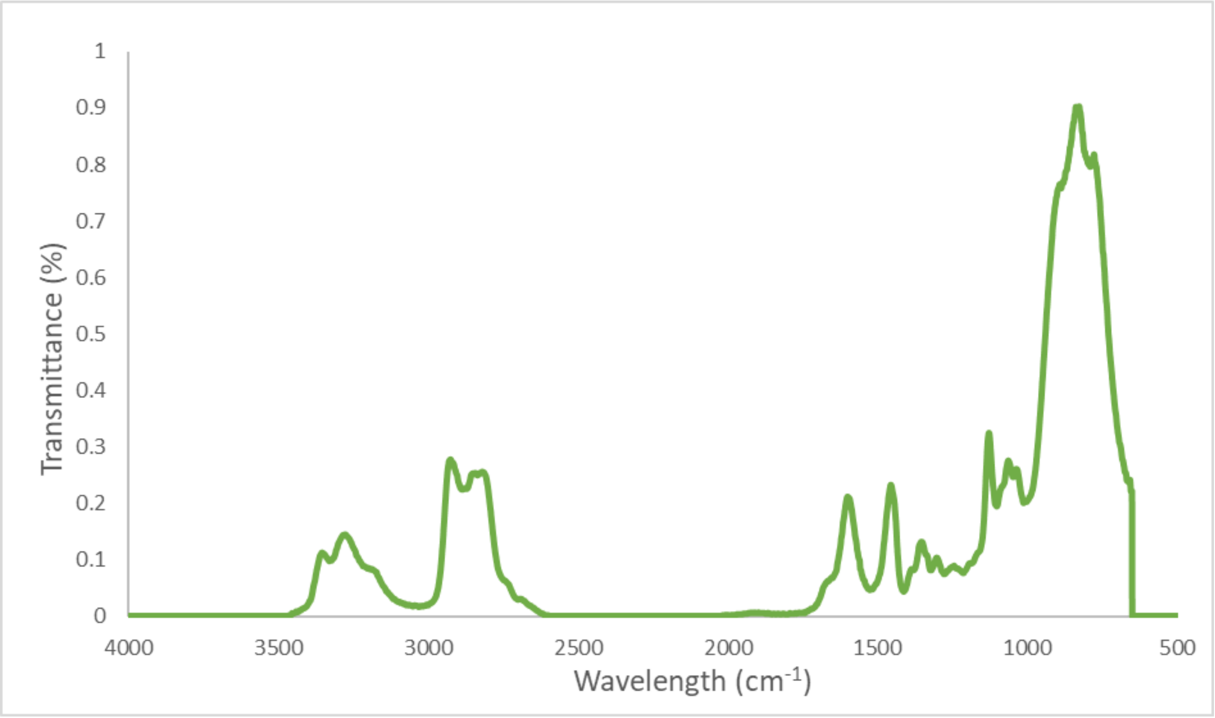

Fig. S42 FT-IR spectra of CSBO obtained using different IL catalysts.

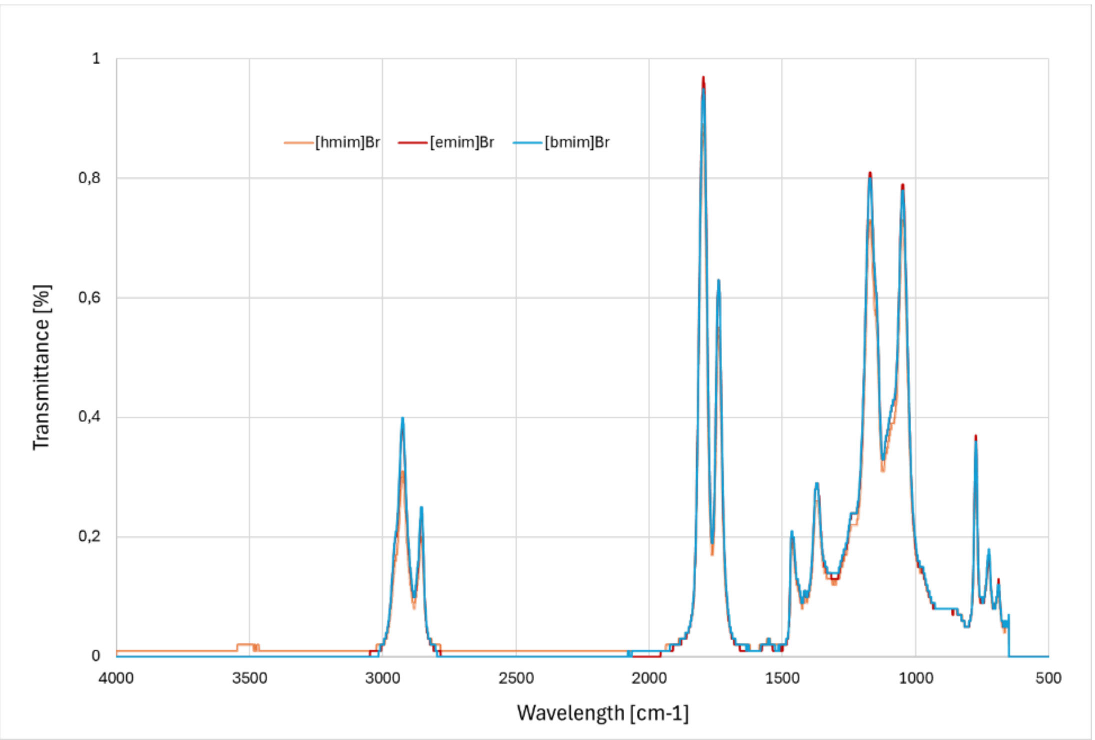

Supplement: Supplementary file 1 [file molecules-29-03908-s001.zip › molecules-3141617-supplementary.pdf]
